# Radiative cooling sorbent towards all weather ambient water harvesting

**DOI:** 10.1038/s44172-023-00082-3

**Published:** 2023-06-09

**Authors:** Wenkai Zhu, Yun Zhang, Chi Zhang, Xiwei Shan, Akshay K. Rao, Sarah L. Pitts, Travest J. Woodbury, Tanya Sophia Masnyk, Dominique Derome, David M. Warsinger, Xiulin Ruan, Lisa J. Mauer, Jan Carmeliet, Tian Li

**Affiliations:** 1 School of Mechanical Engineering, Purdue University, West Lafayette, IN 47907 USA; 2 Chair of Building Physics, ETH Zürich, Zürich, 8092 Switzerland; 3 Department of Food Sciences, Purdue University, West Lafayette, IN 47907 USA; 4 Department of Civil and Building Engineering, Université de Sherbrooke, Sherbrooke, QC J1K 2R1 Canada

**Keywords:** Mechanical engineering, Porous materials

## Abstract

Emerging atmospheric water harvesting (AWH) technologies hold promise for water supply to underdeveloped regions with limited access to liquid water resources. The prevailing AWH systems, including condensation- or sorption-based, mostly rely on a single mechanism limited by working conditions and inferior performance. Here, we synergistically integrate multiple mechanisms, including thermosorption effect, radiative cooling, and multiscale cellulose-water interactions to improve the water harvesting performance with minimal active energy input over a relative humidity (RH) range between 8% to 100%. The proposed system consists of a scalable and sustainable cellulose scaffold impregnated with hygroscopic lithium chloride (LiCl). Cellulose scaffold and LiCl synergistically interact with water at molecular, nanometer, and micrometer scales, achieving a high yield (2.5–16 kg kg^−1^ at 60–90% RH). The captured water in return facilitates radiative cooling due to its intrinsically high infrared emissivity. An outdoor batch-mode AWH device shows a water uptake up to 6.75 L kg^−1^ day^−1^ with a material cost as low as 3.15–5.86 USD kg^−1^. A theoretical model is also proposed to elucidate the synergistic AWH mechanisms among cellulose-LiCl-water-energy interaction. This AWH strategy provides a potential solution to water scarcity problems in regions with larger seasonal and climate variations, especially arid areas.

## Abstract (web summary)

A water harvesting strategy utilizing a hygroscopic lithium chloride impregnated cellulose scaffold yields high water harvesting rate with low energy input over a wide range of relative humidity. The strategy, reported by Wenkai Zhu, Yun Zhang, Chi Zhang and coworkers, provides a potential solution to the global water scarcity problem particularly in arid areas.

## Introduction

About half of the world population, i.e., ~4 billion people, undergo severe water scarcity at least seasonally, posing a systematic threat to humanity^1^. Technologies, such as thermal desalination^2–7^ and solar steam generation^8–10^, have been developed and used to mitigate the global freshwater crisis. However, these conventional approaches demand intense energy input and, most importantly, access to water resources, such as precipitation, surface- or groundwater, or coastal water, limiting their applicability. In search of geographically independent water collection, atmospheric water harvesting (AWH) emerges as a promising candidate to harvest freshwater with drinking water quality and potential scalability.

Atmospheric water vapor and droplets account for ~10% of the global freshwater reservoir^11,12^. There are tremendous efforts in developing AWH systems, most of which can be categorized as either condensation- or sorption-based devices. Condensation-based AWH systems rely on cooling or mechanical compressing to induce vapor-to-liquid phase transition. Their major drawbacks include the requirement for high relative humidity (RH) > 70%^13–16^ or sizeable energy input for cooling^17,18^, which are not viable for arid and developing regions. In contrast to condensation, sorption-based AWH systems can work over a much wider RH range (>10% RH), exploiting the hygroscopicity of sorbents. Typical sorption materials include desiccator salts^19–21^, metal/covalent organic frameworks^22–25^, thermo-responsive gels^26–28^, and carbon-based systems^29^. Strong water affinity contributes to water capture but hampers subsequent water release. Yet to be resolved is the long-standing quest for simultaneous enhancement of water capture and release, two processes difficult to be optimized simultaneously. Also, sorption materials that are fully sustainable, cost-effective, and scalable are lacking. Recently, a radiative cooling effect of cellulose fabric is found to be beneficial to AWH because it facilitates the exothermic sorption process and lowers the saturation vapor pressure which consequently promotes condensation^30^. However, the reported working range and capacity are fundamentally limited by the modest hygroscopicity of cellulose alone. Integrating LiCl into porous frameworks has been a desired option for its high hygroscopicity^31–33^. However, these strategies involve either complicated synthetic processes or limited scalability.

This study aims to tackle the above-mentioned challenges by impregnating LiCl in the cellulose scaffold with a facile dip-coating method to extend the working range and boost the water harvesting ability. This is enabled by the copious hydrogen bonding in the cellulose chain for a rapid formation of hydration shells that allow a simple and stable nanoscale LiCl integration. The proposed strategy also synergistically integrates multiple mechanisms, including the thermosorption effect, radiative cooling, and multiscale cellulose water interaction, forming an integrated protocol that has not yet been explored in full detail. We demonstrate the fundamental interplay of key components in a dual-mechanism AWH system by a LiCl–cellulose radiative cooling composite and document its promising performance at both low and high RH. Moreover, we investigate a self-adaptive switch between water capture and release for multicyclic operations. Lab experiments, field tests, and theoretical modeling are conducted to elaborate on how the microscopic LiCl–cellulose–water coupling and the environmental factors synergistically affect the macroscopic material performance. The theoretical model is cross-validated with the experimental water uptake with or without radiative cooling and predicts water uptake beyond the current experimental limits. An outdoor batch-mode AWH device demonstrates water capture (water uptake) as high as 6.75 L kg^−1^ day^−1^ (70% RH, 21.6 °C) and water production up to 5.97 L kg^−1^ day^−1^ under 0.9–1.1 sun (0.9–1.1 kW m^−2^) after eight continuous capture-release cycles with a material cost as low as 3.15–5.86 USD kg^−1^ (nonwoven fabric as cellulose scaffold: 1.5–2.8 USD kg^−1^, industrial grade LiCl: 7–13 USD kg^−1^). The efficient and high-performance AWH composite operating over a wide range of the RH spectrum opens opportunities for both fundamental research and practical applications to account for the seasonal and climate variation of the RH, especially in arid areas (such as the Death Valley), where RH varies between 5% and 95% most years.

**Fig. 1:**
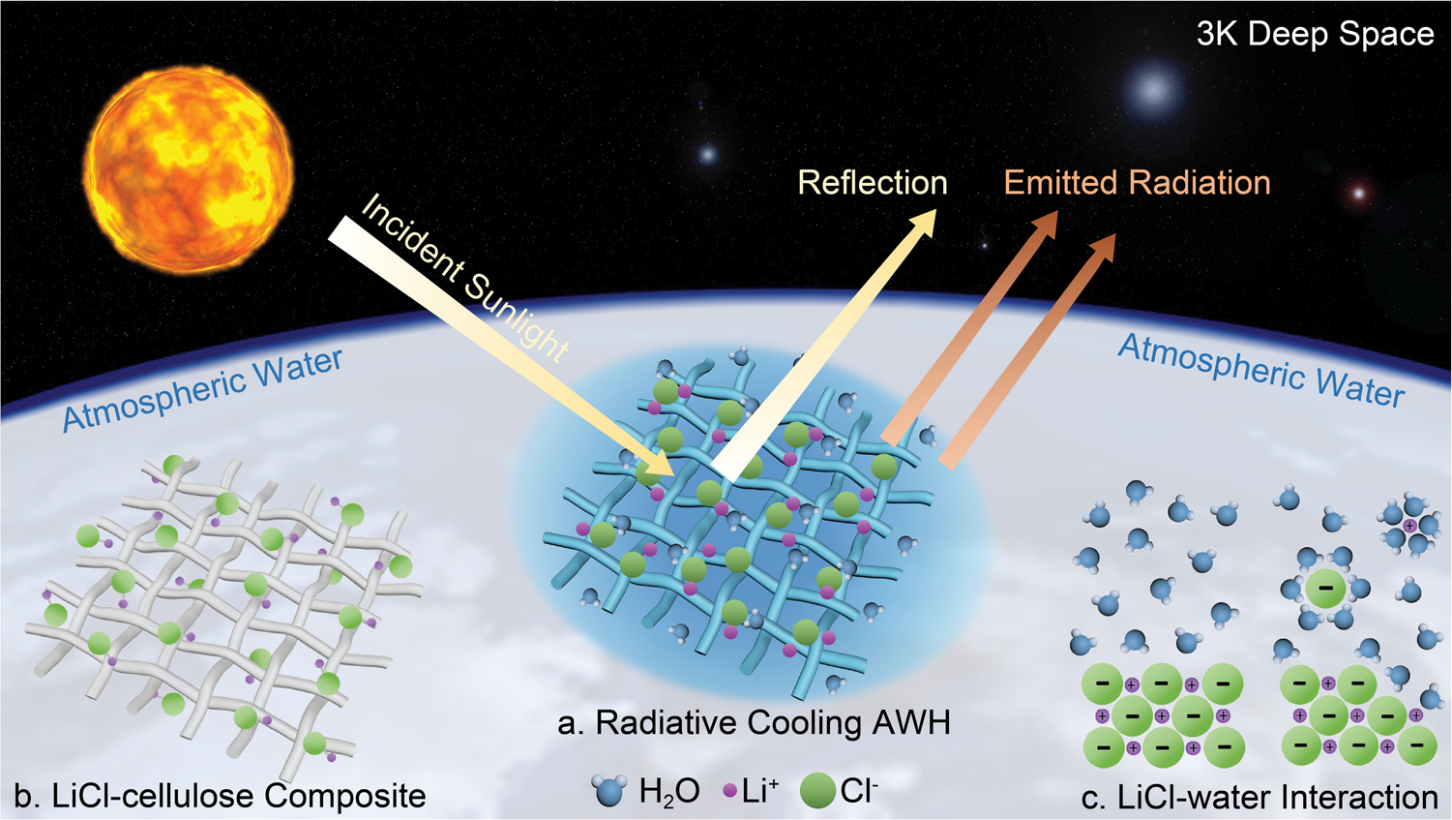
LiCl–cellulose composite demonstrates energy-efficient atmospheric water harvesting. **a** Schematic of mechanisms of atmospheric water harvesting by radiative cooling LiCl–cellulose composite. **b** Scaffolds of LiCl–cellulose composite and complex networks show strong physical entanglements and water storage capabilities. **c** Molecular interaction between LiCl and water.

Cellulose, one of the most abundant biopolymers on earth, has many unique functional properties including robust mechanics, radiative cooling effect, strong hydrophilicity, carbon-neutrality, etc.^34–37^. Radiative cooling and sorption form a positive feedback loop (Fig. 1a). Cooling promotes sorption while the adsorbed water molecules contribute to an even higher cooling power due to their high infrared (IR) emissivity. LiCl salt adsorbs limited amounts of water at low RH conditions (<5% RH), but when the RH is raised above 11%, the deliquescence point (RH_o_) of LiCl, the salt will deliquesce, forming a saturated solution that can ultimately take up more than 1000% water content as the RH is elevated further^38,39^. LiCl desiccant is also advantageous in terms of water release, as LiCl regenerates at 40 °C^40^, a temperature readily obtainable via solar heating. Impregnation of LiCl in the cellulose multiscale hierarchical pore network extends the working RH range and boosts the water harvesting capacity and kinetics, compared to salt-alone and cellulose-alone systems. The cellulose scaffold, in return, structurally supports LiCl and retains the water and LiCl solution (Fig. 1b). The aggregation, agglomeration, and deliquescence commonly observed in LiCl powder (Fig. 1c) are alleviated in the cellulose scaffold for a non-degraded sorption capability^20,39,41–43^. The hydrated LiCl–cellulose composite increases optical transparency across the solar spectrum and allows rapid water evaporation by solar heating with a dark substrate underneath. Cycles of radiative cooling and solar heating shift the system temperature between low and high favoring water capture and release, respectively. This yields a high harvesting capacity which has been one of the major bottlenecks in conventional ambient water harvesting systems.

## Results and discussion

### Synthesis and morphology

**Fig. 2:**
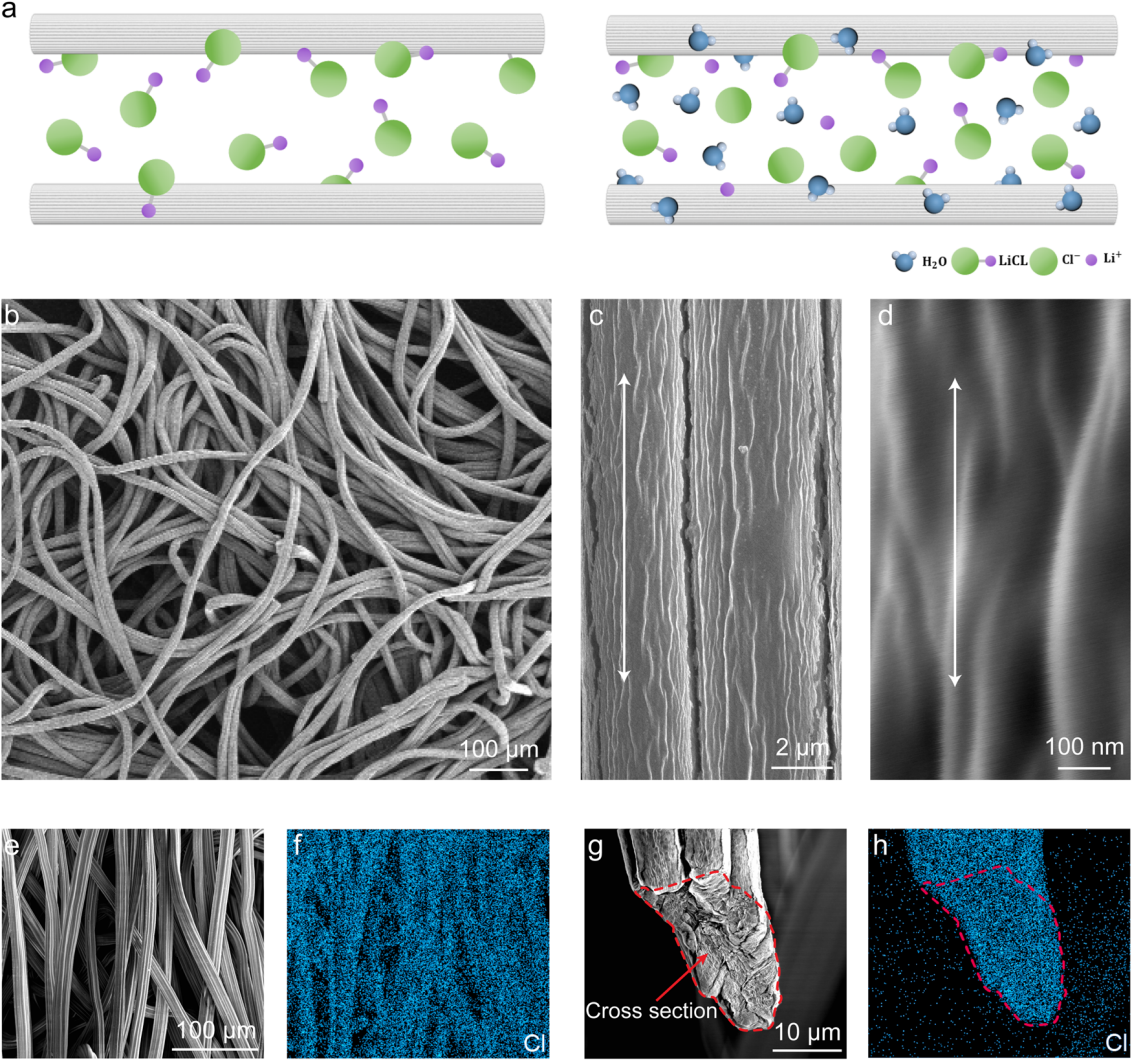
Synthesis and morphology of the LiCl–cellulose composite. **a** Schematic of the LiCl ion intercalation process. **b** SEM of LiCl–cellulose composite composed of entangled cellulose fiber bundles. **c** A magnified view of the cellulose fiber surface. **d** Partially aligned cellulose nanofibers. **e** Cellulose fiber network. **f** The corresponding EDS mapping of Cl shows the coating uniformity. **g** Cross-section view of the cellulose fiber bundle by severing the fibers after the infiltration process. **h** The corresponding Cl mapping demonstrates that LiCl is impregnated into the fibers by intercalation.

To fabricate the LiCl–cellulose composite, the cellulose scaffold (0.574 mm thickness fabric) is immersed in LiCl solution and then oven-dried. The LiCl ions gradually intercalate into the cellulose fiber matrix during three times of dipping-drying steps (Fig. 2a, Fig. S1). Following the oven drying process, the well-dispersed ions recrystallize and bind tightly to the cellulose surfaces. Fabrication details are given in the “Methods” section. The high aspect ratio of the fiber bundle is composed of multiscale features, including microscale fiber bundles ~20 μm in diameter to nanofibers ~20 nm in diameter (Fig. 2b–d, Fig. S2). The LiCl brine solution quickly infiltrates the cellulose scaffold, and the salt ions achieve nanoscale dispersion, as indicated by the energy-dispersive X-ray spectroscopy (EDS) mappings of fiber networks and cross-sections (Fig. 2e–h, Figs. S3 and 4). More details of EDS analysis can be found in Note S1. The hydrophilic surface as well as the numerous channels between fibers and fiber bundles, revealed in the scanning electron microscope (SEM) images (Fig. 2b–h), facilitate the salt solution infiltration process. The intrinsically hydrophilic and highly porous fiber network efficiently captures and stores water. Also, the LiCl–cellulose composite exhibits higher mechanical strength than the cellulose scaffold in the dry condition in Fig. S5, attributed to the fiber’s aggregation after the soaking and drying process, while the difference between their mechanical strengths is hardly observed in the wet condition. Our solution-based salt impregnation process has an estimated cost of 0.297 USD m^−2^ (Note S2) and can be easily incorporated into the well-established cellulose-based paper, textile, and membrane manufacturing process.

### Optical and thermal characterization

**Fig. 3:**
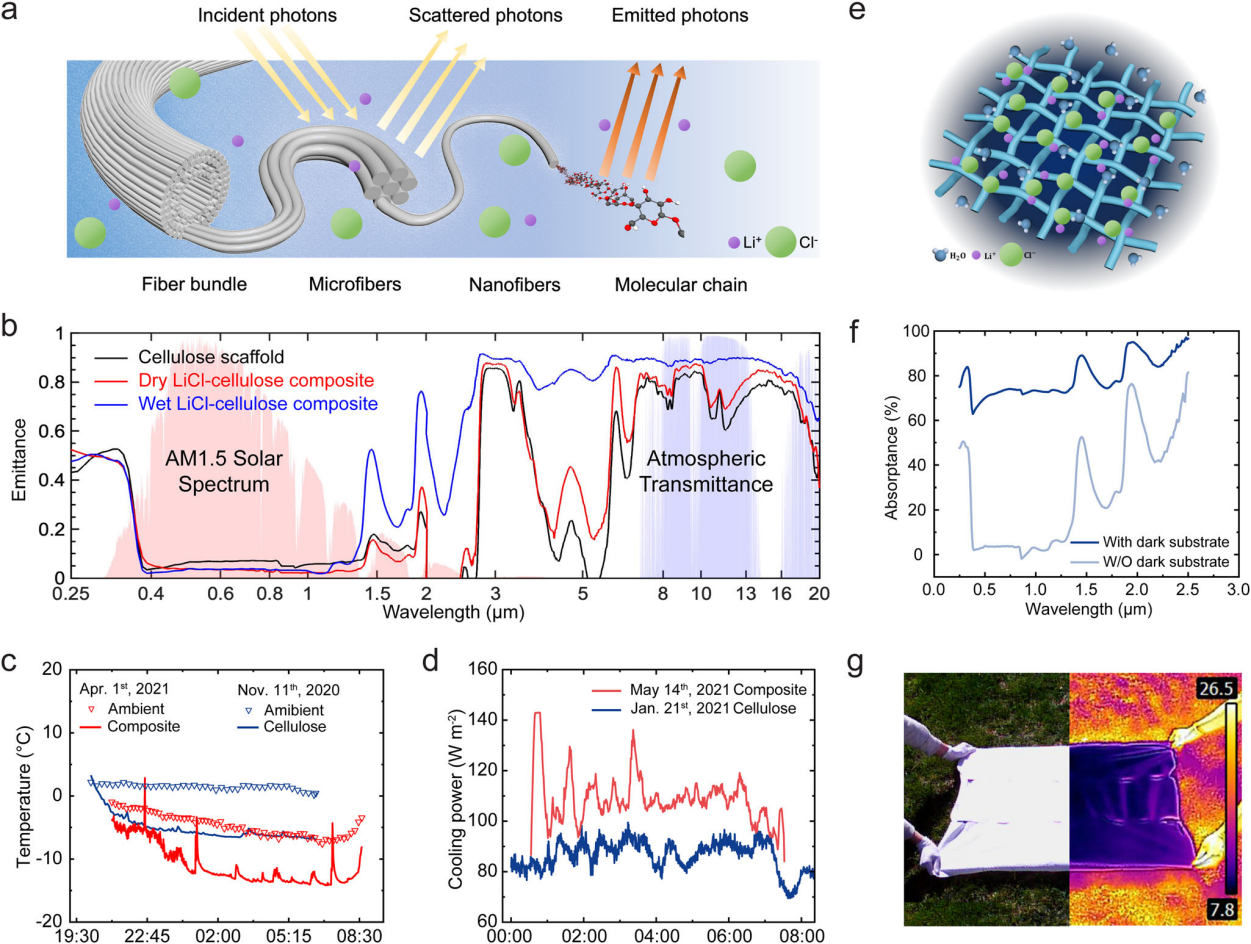
Optical and thermal characterization of LiCl–cellulose composite. **a** Schematic of the principle of radiative cooling effect by the LiCl–cellulose composite. **b** Emittance in the ultraviolet, visible, and infrared range of the cellulose and LiCl–cellulose composite in dry and wet conditions. **c** Profiles of the sample surface temperatures and ambient temperature of cellulose scaffold and LiCl–cellulose composite on two different nights. **d** Profiles of the cooling power measured for the cellulose scaffold and LiCl–cellulose composite on two different nights. **e** Schematic of water release from the LiCl–cellulose composite with an underneath dark substrate. **f** Absorptance of LiCl–cellulose composite with and without dark substrate. **g** Macroscopic and IR photo of a large-scale LiCl–cellulose composite on April 30th, 2021. The ambient temperature was 21 °C.

Radiative cooling is a systematic outcome of photonic and vibrational interactions between the multiscale structure of cellulose, solar radiation, and the ambient environment (Fig. 3a). Figure 3b exhibits the emittance over the wavelength range 250 nm–20 μm, i.e., solar spectrum and sky transmittance spectrum (IR range), of the cellulose scaffold and LiCl–cellulose composite in dry and wet states. The transmittance of the LiCl–cellulose composite indicates that 50.7% of solar radiation passes through the sample (Fig. S6), while the presence of water increases the wet-state transmittance by 13.5%. The reflectance of the composite sample in the solar spectrum reduces to 17.3% in the wet state (Fig. S7). In the visible and near-IR spectra, the emittance is found to be low because incident radiation with a wavelength between 0.4 and 1.5 μm is intensely scattered by the nanofibers and microfibers at the corresponding length scale. The particularly strong IR radiation of cellulose stems from the multimode vibrations of molecular chains, including C–O stretching and O–H stretching^44^, where the emission wavelengths align with the atmospheric sky window (8–13 μm), permitting energy dissipation to deep space. The microfibers and larger fiber bundles (2–20 μm) contribute to amplifying and broadening the IR emittance peaks to increase the radiative energy leaving the composite. The aforementioned light–matter interactions provide the LiCl–cellulose composite a selective emittance spectrum that allows a spontaneous and rapid cooling to sub-ambient surface temperature during nighttime^45^. Wet samples emit more strongly in the IR range than dry ones because the additional hydrogen bonding between the OH^−^ branches and H_2_O molecules complements the missing molecular vibration modes, and water occupies the original pore space providing more emitting substances. Also, bulk water itself is a good IR emitter with near-unity emissivity^46^. These suggest that sorption and radiative cooling mutually enhance each other, forming a positive feedback loop as a merit of system design. The effective emissivity over the atmospheric window is 0.75 for the cellulose scaffold in the dry condition and is enhanced to 0.80 when coated with LiCl.

Field tests are conducted to demonstrate the functionality of the AWH composite in real environments. The field test procedure and apparatuses are illustrated in the “Methods” section and Fig. S8, respectively. Water harvesting tests were carried out during nights from Nov. 2020 to May 2021 in West Lafayette, IN (40.4237° N, 86.9212° W). Radiative cooling effectively reduced sample surface temperature to 6.0–8.7 °C below ambient temperature, as shown by the representative data of April 1st, 2021, and November 11th, 2020, at an RH range of 48–82% (Fig. 3c). The sporadic temperature surge in Fig. 3c was caused by the manual measurement of the sample weight. The radiative cooling power measured by a feedback heater (Fig. 3d) exhibited an average cooling power of 110 W m^−2^ for LiCl–cellulose composite during the night of May 14th, 2021, and 82.3 W m^−2^ for the cellulose scaffold during the night of January 21st, 2021. The cooling power difference was a consequence of different climate conditions. Although water capture by sorption or condensation was exothermic, our estimation indicated that the radiative cooling effect was not disturbed by the water-capturing process (Note S3). The LiCl–cellulose composite showed a higher cooling power when the test was conducted under a higher ambient temperature and low absolute humidity during the summer. We present a theoretical model to estimate the cooling power at different ambient temperatures based on the measured optical emittance of the three samples (Note S4). The wet composite shows the highest theoretical cooling power, while the dry cellulose scaffold has the lowest one (Fig. S9). Moreover, in the wet state, the LiCl–cellulose composite can transmit more sunlight because of a better match of the refractive indices of cellulose and water than cellulose and air (Fig. S6). Thus, a dark substrate can be used underneath the wet composite to improve the solar absorption and raise the sample temperature to facilitate water release (Fig. 3e). During desorption, rapid water release can be achieved via a solar absorption of 67.7% by the dark substrate during the daytime (Fig. 3f). The numerous channels and fiber surfaces serve as paths for water transport from inside to the composite surface enhancing the drying process and release of stored water^34^. The outdoor cellulose scaffold demonstrated a lower surface temperature of the composite due to radiative cooling, with exposure to direct solar irradiation, as shown in Fig. 3g.

### Synergistic mechanisms

**Fig. 4:**
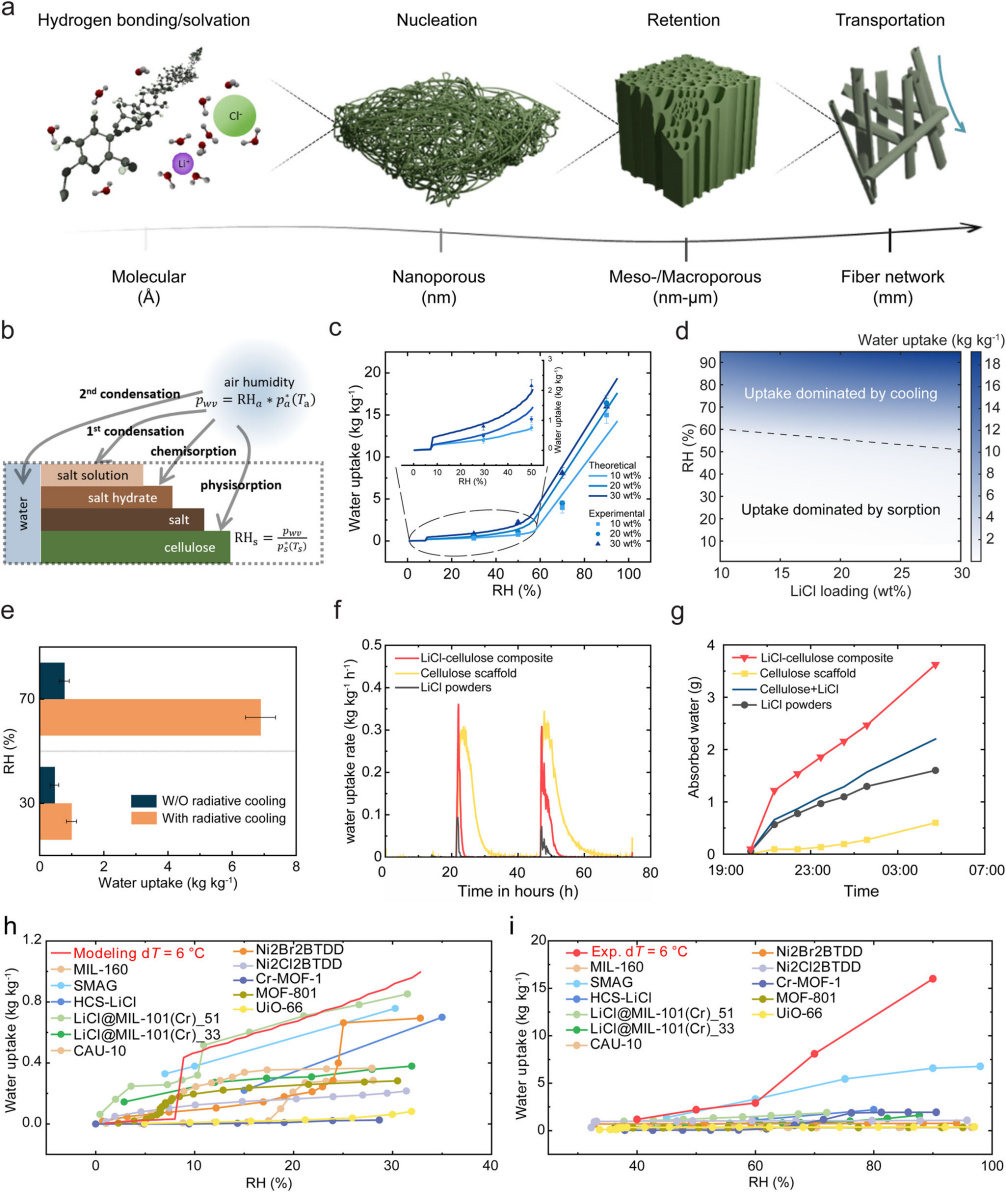
Synergistic effects among radiative cooling, hygroscopic LiCl, and cellulose scaffold. **a** Schematic of multiscale interaction between water and LiCl–cellulose composite system. **b** Schematic of four sorption mechanisms for theoretical modeling. **c** Experimental and theoretical water uptake of LiCl–cellulose composite samples with 10–30 wt% at 0–100% RH. Inset is an enlargement of the experimental and theoretical water uptake from 0–50% RH. **d** Water uptake as a function of RH and LiCl loading for samples at 6 °C below the 25 °C ambient temperature. **e** Water uptake comparison of 30 wt% LiCl–cellulose composites with and without radiative cooling effect at 30% and 70% RH. The error bars of **c** and **e** represent the propagated errors from mass measurement. **f** Rate of water uptake of cellulose, LiCl, and LiCl–cellulose composite samples based on sorption isotherm characterization. **g** Collected water mass as a function of time during the field test of cellulose, LiCl, and LiCl–cellulose composite. The uncertainty is 0.01 g. Water uptake comparison with other sorbents for AWH at the lower end (**h**) and the higher end (**i**) of the RH spectrum. d*T* is the sample temperature below the ambient temperature.

To further elucidate the working mechanism of the LiCl–cellulose composite, we individually analyze and decouple the synergistic effect of the radiative cooling, hygroscopic LiCl, and cellulose fibril structure (Fig. 4a). At the atomistic scale, the water dipole attracts the counter-ions and breaks the ionic bonds of LiCl leading to its dissolution. Meanwhile, the polar hydroxyl groups on the cellulose chain hydrogen bond strongly with polar water molecules. The multiscale pore structure of the cellulose scaffold facilitates water harvesting with micropore filling (<2 nm), mono/multilayer adsorption on pore surfaces, and capillary condensation in mesopores (>4 nm). In nanoscale pores, the intermolecular interactions of the surfaces overlap, thus creating strong attraction-promoting nucleation. The adsorption can occur even at low RH. The fibers at this scale are cellulose aggregates. On the mesopore scale, a large volume of water is retained via capillary forces. Capillary condensation happens at sub-saturation vapor pressure, as described by the Kelvin equation. The porous structure at this scale denotes larger-scale fiber assemblies. Larger scale structures, such as the fiber network, transport water by wicking in the inter-fiber pores spaces, as also occurs in desert plants^47^. Single fibers consist of a bundle of cells. The enhanced water-capturing performance stems from four mechanisms (Fig. 4b): 1. Physisorption of cellulose; 2. Chemisorption of LiCl, i.e., forming LiCl hydrates; 3. Sorption of water vapor into LiCl solution (here called first condensation) occurs when RH is higher than the RH_o_; 4. Condensation of water vapor due to vapor saturation (here called second condensation). Each of these mechanisms can be described by corresponding physical models, where the details can be found in Note S5. The physisorption of cellulose is frequently represented by the Guggenheim–Anderson–de Boer (GAB) sorption model, which describes type II sorption behavior with monolayer water uptake and energy terms of free enthalpy differences. The hydration of LiCl can be described by its phase diagram^38,48^. Isolated water condensation (second condensation) is a (quasi) linear function of time, assuming heat and mass transfer reach equilibrium. With these considerations, a theoretical model for the sorption of the composite is developed as the weighted summation of the four mechanisms (Note S5).

Controlled experiments are conducted in an environmental chamber (Fig. S10) with details in the “Methods” section. The working mechanisms are identical to the field test but with precise control of the sample temperature, ambient temperature, and RH for a systematic performance evaluation. Figure 4c displays the water uptake curves at a 6 °C temperature drop from 25 °C ambient temperature, 10–30 wt% LiCl content. At ~8% RH, sorption surges due to the onset of salt deliquescence and the increase of effective RH by radiative cooling (inset of Fig. 4c). Figure S11 shows similar water uptake profiles but with a constant 9 °C below the ambient temperature. The higher water uptake profile is attributed to the enhanced cooling effect, which can be achieved by reducing the convection loss or adding an IR emissive substrate underneath that further maintains a lower sample surface temperature. Sorption dominates the water uptake until RH > 60%, where condensation commences and dominates. The theoretical model quantitatively agrees with the experiments, as shown in Fig. 4c. The outcome of this model also clearly demonstrates that radiative cooling substantially improved the water capture (Fig. S12). Furthermore, the model allows us to derive water uptake based on LiCl content and RH, as shown in Fig. 4d. The diagram breaks down into two regimes where one of the two mechanisms dominates performance. In sub-dew point temperature conditions, water capture is driven by the radiative cooling power to cool the scaffold compensating for the latent heat transfer due to condensation. Whereas above the dew point, it is sorption driven, and the enhancement comes from the increase of internal RH in the LiCl–cellulose composite by radiative cooling. The lab tests reveal a 2-fold and 9-fold increase in water uptake solely due to the 6 °C cooling of the composite at 30% and 70% RH, respectively (Fig. 4e). The effect of radiative cooling on the water uptake is more pronounced at higher RH since condensation starts to dominate the water capturing. A comparative field test conducted using two pieces of 30 wt% LiCl–cellulose composite, with one exposed to the sky and the other shielded by non-transmissive cardboard (Fig. S13a), also shows the radiative cooling effect on the enhancement of water uptake (Note S6, Fig. S13b).

In addition to the synergistic thermosorption effect, which enhances water capture capacity at lower temperatures, water capture kinetics is also greatly expedited by dispersion, as indicated by the dynamic vapor sorption experiments (see “Methods” section, Note S7) and field tests. More specifically, in terms of the rate of water uptake, LiCl–cellulose composite has a synergistically higher rate of water uptake and outperforms the summation of LiCl powder and cellulose scaffold alone. We employ nitrogen sorption isotherms to determine the surface area of LiCl, cellulose scaffold, and LiCl–cellulose composite. The LiCl–cellulose composite has a Brunauer–Emmett–Teller (BET) equivalent surface area of 0.331–0.366 m^2^ g^−1^, while the values of cellulose scaffold and LiCl powders are 0.177 and 0.047 m^2^ g^−1^ respectively (see “Methods” section and Note S8). The impregnated LiCl is dispersed and attached to the large surface area of the cellulose scaffold, resulting in a much greater surface area for the composite that enhances sorption kinetics. The rates of water uptake of the three samples, based on the derivative of the water uptake vs. time curve without radiative cooling effect according to vapor sorption experiments (Note S7, Fig. S14), exhibit the rapid saturation and high kinetics of LiCl–cellulose composite’s sorption behavior (Fig. 4f). Also, the LiCl–cellulose composite absorbed more water compared to the sum of the cellulose scaffold and the LiCl powder in the field test (Fig. 4g). The results in Fig. 4f, g support our hypothesis that the synergistic effect in the composite not only enhances the water capture capacity but also accelerates the water capture kinetics. Comparisons in Fig. 4h, i show the developed LiCl–cellulose composite has competitive water uptakes compared to reported AWH sorbent materials under most weather conditions (8–90% RH).

### Performance validation and impact

**Fig. 5:**
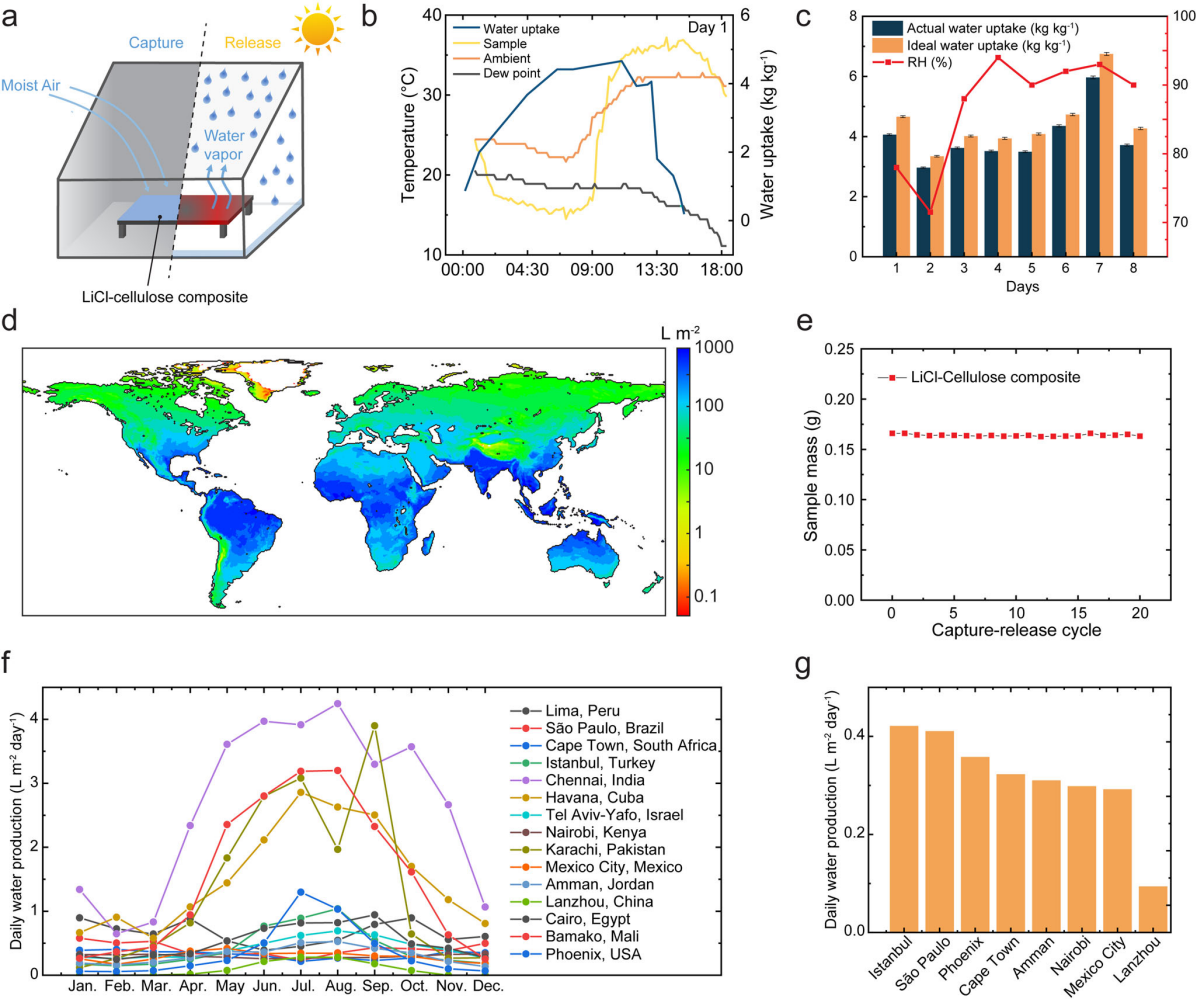
Scalability, impact, and the prospect of LiCl–cellulose composite for atmospheric water harvesting. **a** Schematic of the AWH device with the LiCl–cellulose composite. **b** Temperature and water uptake of a 30 wt% LiCl–cellulose composite during a field test. **c** Field test results of 8 days, including ideal and actual water uptake in kg kg^−1^. The actual water uptake is defined as the mass of water released over the initial mass of the composite. The ideal water uptake is defined as the mass of water captured over the initial mass of the composite. The error bars represent the propagated errors from mass measurement. **d** Predicted annual water collection per area based on local climate around the globe. **e** Dry mass of a 30 wt% LiCl–cellulose composite during 20 capturing-releasing cycles, which exhibits the LiCl is stable and will be retained in the composite for a durable application. The uncertainty is 0.0001 g. **f** Estimated average monthly water production in arid cities around the world. **g** Average water production for the eight most arid cities from (**f**).

Outdoor experiments are conducted under natural sunlight to characterize system performance in real-life scenarios. A batch-mode AWH prototype consisting of a thin-wall and clear acrylic container and a LiCl–cellulose composite with a dark substrate was assembled (Fig. 5a, Fig. S15). During the nighttime, the lid of the acrylic container was opened, exposing the LiCl–cellulose composite (approximate dimension 10 × 15 × 0.1 cm^3^, mass 1.5136 g) to the sky, and the composite captured water from the ambient air. During the daytime, the container was sealed. Driven by solar heating, the water captured during the night evaporated and continuously condensed on the inner wall of the acrylic as the composite temperature increased. Then the liquid water flowed down along the slope of the top container and accumulated in the corner.

An 8th-day AWH was demonstrated on June 13th–14th and July 2nd–July 7th, 2021. Figure 5b exemplifies one day of water harvesting (June 13th), where the ambient temperature, sample surface temperature, and dew point were monitored. The water uptake of LiCl–cellulose composite consistently increased during the entire capturing process. The actual water uptake on the first day reached 4.06 kg kg^−1^, 13% lower compared to the ideal water uptake. Sunrise began at 6:16 AM, the outdoor temperature quickly increased from 22 °C to 28 °C, and the RH decreased from 78% to 59%. At 11:03 AM, the acrylic container was sealed, and the water-releasing process started. Within 1 min, the inner wall of the device started to mist, and the transparent container became translucent after 5 min. The evaporated water gradually condensed on the inner wall of the device (Fig. S15b). Between 11:03 AM and 3:24 PM, the device harvested 3.65 kg kg^−1^ of liquid water, which was 86% of the captured water in the LiCl–cellulose composite. Figure 5c demonstrates the repeatability of the liquid water collection in the field test on the next seven consecutive days, where the highest water uptake was 5.97 kg kg^−1^ on July 6th.

The element analysis by ICP-MS shows that the quality of the collected water complies with the WHO standard (Note S9, Fig. S16). Capture-release cycles are repeated 20 times in lab tests. The water release is initiated by a solar simulator (Note S10, Fig. S17). The composite is shown to be durable without notable degradation, as revealed by the stable weight of the LiCl–cellulose composite in an accelerated cyclic test (Fig. 5e, Fig. S18). Above material characterizations and performance demonstrations confirm that the LiCl–cellulose composite is a potential candidate for achieving efficient AWH due to its distinct advantages of high water sorption capacity at low humidity, fast water sorption–desorption kinetics, continuous sub-ambient cooling, and stable cyclability. With a comprehensive understanding of the mechanisms and performance of LiCl–cellulose composite, especially its noticeable portability, scalability, and high water uptake, we then model and predict the potential daily water production on a global scale using the performance characterized in the lab test. The modeling takes the global climate data from the national centers for environmental prediction (NCEP) for the year 2021 and estimates the water production rate based on the local RH, ambient temperature, and total cloud coverage (Note S11). With the consideration of key ambient factors, there is still a non-zero annual production of clean water even in the most draught region. The annual cumulative water production solely by AWH can reach 1000 L m^−2^ in some insular and peninsular areas where the humidity level is high, but the freshwater resource is scarce (Fig. 5d). The average daily water production in typical arid cities from Jan. to Dec. is shown in Fig. 5f, g. As climate conditions differ city by city, the LiCl–cellulose composite can harvest four times more water from the atmosphere in Istanbul than in Lanzhou. The high-performance LiCl–cellulose composite has the potential to mitigate the water shortage and the associated global challenges.

## Conclusion

We demonstrate a full functionality and high-efficiency AWH by the passive radiative cooling LiCl–cellulose composite working across a wide range of RH (>8% RH, 25 °C) and elaborate the synergistic integration: (1) dispersed hygroscopic LiCl for moisture sorption and liquefaction, (2) multiscale cellulose structure for water storage and transport, (3) passive radiative cooling enhanced sorption and condensation with minimal active energy consumption, and (4) improved solar absorption by a dark substrate for rapid water release. The established and validated theoretical model involving different sorption mechanisms and the radiative cooling effect well predicts the experimental water uptake and explains the synergy among the composite-water-energy interaction. Field tests demonstrate a water production rate of up to 5.97 L kg^−1^ day^−1^ under 0.9–1.1 sun (0.9–1.1 kW m^−2^). These synergistic functions render an extraordinarily promising and scalable approach for an AWH system with a high production rate, cost-effectiveness, environmental friendliness, high stability, and completely safe water collection. The rational system design utilizing the multiscale structure of cellulose and the synergistic effect of different mechanisms paves the way for future advanced AWH systems.

## Methods

### Fabrication of LiCl–cellulose composite

To prepare the different LiCl loadings on the cellulose sample, we dissolved LiCl crystals (Sigma Aldrich) into 200 ml of deionized water to obtain 0.15, 0.3, and 0.5 mol L^−1^ LiCl solutions. Cellulose samples were pre-dried at 80 °C for 30 min, and the dry mass of the cellulose sample was measured. Then the sample was immersed into the LiCl solution and oven-dried at 80 °C for 20–40 min, depending on the sample size (Fig. S1). The immersing-drying process was repeated three times. The final mass of the composite sample was measured, and the LiCl loading weight percent (wt%) was obtained. The LiCl–cellulose composites were stored in sealed containers and dried for 5–10 min in an 80 °C oven before further testing. The area density of the cellulose scaffold is 0.0416 kg m^−2^. Dividing it by the cellulose content of LiCl–cellulose composite (1 − LiCl wt%) gives the area density of the composite for unit conversion of water uptake between kg kg^−1^ and kg m^−2^.

### Mechanical test

The MTS Criterion^®^ Series 40 Electromechanical Universal Test System was used as a tensile meter to measure the mechanical tensile strength and toughness of the cellulose samples. The LiCl–cellulose composite and cellulose in both wet and dry states were cut into slices of 1 × 5 cm size. A parallel load to the sample’s plane direction was applied, while the elongation was recorded at the same time during the test. The load over the cross-sectional area of each sample slice was used to calculate the nominal tensile stress. For each slide, optical microscopy was used to get the average width and thickness to calculate the cross-sectional area. The displacement over the original length of the sample slice’s elongated part was used to calculate the strain. The maximum value of the strain-stress curve was considered as the strength.

### Optical spectrum characterization

The total hemispheric transmittance and reflectance from 0.25 to 2.5 μm were measured by a Perkin Elmer Lambda 950 UV–VIS–NIR spectrometer (±0.02% uncertainty) with an integrating sphere and a certified Spectralon diffuse reflectance standard. Additionally, a Nicolet iS50 FTIR spectrometer with a PIKE Technologies integrating sphere was used to measure the total hemispheric transmittance and reflectance between 2.5 and 20 μm with ±0.5% uncertainty. Kirchhoff’s law and the thermal equilibrium principle were used to derive the emittance spectrum.

### Field test of water uptake and temperature

An insulated plastic table was used as a platform to support the samples with a length of 150 mm and a width of 130 mm. To ensure a fair comparison, the sum of the dry mass of the cellulose scaffold and the LiCl powder was close to the dry mass of the LiCl–cellulose composite. The field tests were conducted during the winter and spring, and the test location is 40.4237° N, 86.9212° W in West Lafayette, IN. For every 2 h, a balance with a precision of ±0.01 g was used to measure the mass of each sample. The water uptake is calculated as the mass of captured water (difference between wet mass and dry mass) over the dry mass. The dry mass was measured before the test and after half-hour oven drying at 80 °C. A local weather website provided the ambient conditions, which were recorded as ambient temperature, RH, wind speed, and dew point every 10 min during the field test. To reduce the convective heat loss, a windshield made of polyethylene foil was adopted. The ambient temperature was monitored by two thermocouples, while the sample temperature was monitored by two T-type thermocouples attached to the top surface of each sample.

### Field test of cooling power

The cooling power measured in the field test was done by a feedback heater (a polyimide resistive heater) that located under the sample and maintained at the ambient temperature (Fig. S8b). A sample size of 50.8 × 50.8 mm was used. The power output of the heater was regarded as the cooling power of the sample.

### A lab test of water uptake

The analogical experiments were conducted in an environmental chamber (ESPEC test chamber) with controlled temperature and RH. A Laird thermoelectric (TE) device of 52 × 52 mm was used to simulate the radiative cooling effects by providing effective cooling to the composite. The TE module was used to maintain the composite temperature at 6 and 9 °C below the chamber temperature according to the results in the field tests shown in Fig. 3c. A 50 × 50 mm LiCl–cellulose composite sample (10, 20, or 30 wt% LiCl) was cropped and tested under 30%, 50%, 70%, and 90% RH at 25 °C ambient temperature, which covered most outdoor conditions. The LiCl–cellulose composite was weighed on a balance with a precision of ±0.01 g after 2-h water adsorption. The water uptakes were calculated based on the initial and final mass. The water uptake measurements of the 30 wt% LiCl–cellulose composite were repeated at 30% and 70% RH.

### Cyclic water harvesting test

To test the sample durability and stability under cyclic water capture and release, a 30 wt% LiCl–cellulose composite was prepared. The absorption was accelerated by a nebulizer to create a highly humid condition, and the desorption was accelerated by an 80 °C hot plate.

### Sorption isotherm by dynamic dewpoint isotherm (DDI)

During the pre-test, the samples (~0.5 g) were maintained at below 5% RH for 6–14 h before the measurement started to remove excessive absorbed water in the samples. The weight at the end of the pre-test was regarded as the reference for water uptake calculation. At constant 25 °C, the RH was monitored when it rose from 5% to 90% dynamically, and the sample weight was recorded by the internal high-precision balance. The water uptake vs. RH for 10–30 wt% LiCl–cellulose composite samples were plotted in Fig. S19.

### Sorption by dynamic vapor sorption (DVS)

DVS measurement was conducted by the Projekt Messtechnik SPS moisture sorption analyzer. All samples (each specimen is ~0.5 g) were kept at 0% RH for 24 h to remove adsorbed/absorbed water from the samples and obtain the dry weight. Then, the samples were subject to 24-h 30% RH and 24-h 70% RH, all at a constant 25 °C. The two test conditions (30% and 70% RH) were selected to represent the lower and higher end of the operating condition for AWH. Measurement of the sample weights occurred every 15 min, and each test condition was held for 24 h to ensure reaching equilibrium.

### True density measurement

The true density of the LiCl–cellulose composite was measured by a gas displacement pycnometer (Micromeritics AccuPyc II 1340). The sample was sealed in a 3.5 cm^3^ instrument compartment. Helium gas was expanded into the compartment and rapidly filled the pore volume. The equilibrium pressures of filling the sample chamber and discharging into a second empty chamber allowed the computation of the sample solid phase volume. The true density was then the sample mass divided by its solid phase volume. Three LiCl–cellulose composite samples of different LiCl loadings were prepared using the dip coating. A pure nonwoven cloth sample was prepared to represent 0 wt% cases. All samples were cropped into small pieces of ~0.5 × 0.5 cm^2^. Each sample of ~0.125 g was measured fives time, and the result was summarized in Fig. S20.

### BET equivalent specific surface area and porosity

The specific surface area was characterized by gas adsorption with Micromeritics TriStar II 3020 to illustrate the impact of the additional LiCl on the effective sorption surface area. The principle of the instrument is by measuring the sorption isotherm of the material, various theories and models can be applied and fitted to generate specific surface area and porosity based on the amount of gas adsorbed on the specific surface. The samples were cooled to the boiling point (77.3 K) of the adsorbate (N_2_) to ensure enough adsorption. The pure nonwoven cloth and 24% LiCl–cellulose composite samples of 0.25–0.4 g were loaded into 3/8 in. tubes. The degassing process was kept at 90 °C for 13 h to remove natively adsorbed water molecules. The sample mass was recorded with an accuracy of 0.0001 g. After the tube was installed and sealed onto the instrument, the samples underwent another degassing and vacuum. The data points were taken for P/P_0_ from 0.001 to 0.3 in a geometric series favoring the lower end of the relative pressure. The quantity of Nitrogen adsorption on the sample surface was recorded as the relative pressure ramped. The BET method fits data in the range of 0.05–0.35 relative pressure, and the slope and intercept of fitting provided the amount of monolayer N_2_ adsorption to derive the BET equivalent specific surface area. Therefore, the results are dependent on the data being fitted. The key criteria of data selection are the intercept being greater than zero and the correlation coefficient *R*^2^ is better than 0.999 for a valid BET fitting. The BET results show the ambient free space volume of both the cellulose scaffold and LiCl–cellulose composite are 37.5 and 24.1 cm^3^ g^−1^, respectively, which are sufficient to retain the amount of water captured.

### Supplementary information

Supplementary Information

## Data Availability

The original data that support the findings of this study are available from the corresponding author upon request. The global climate data used for Fig. 5d can be found at 10.5065/D61C1TXF.

## Code availability

The code for the theoretical cooling power model and theoretical water uptake model can be made available upon request.
