# Supplementary material for: Radiative cooling sorbent towards all weather ambient water harvesting
Source: Commun Eng. 2023 Jun 9;2:35. doi: 10.1038/s44172-023-00082-3 (PMC10955850; doi:10.1038/s44172-023-00082-3)
Supplement: Supplementary file 1 — Supplementary Information [file 44172_2023_82_MOESM1_ESM.pdf]

Supplementary Materials for

Radiative Cooling Sorbent towards All Weather Ambient Water Harvesting

Table of content

Part I: Supplementary Discussions

Note S1. Morphological analysis of LiCl-cellulose composite
Note S2. Cost analysis of the sample preparation
Note S3. Energy consumption estimation for measured water uptake
Note S4. Theoretical cooling power estimation
Note S5. Composite-water-energy interaction mechanism
Note S6. Field test of radiative cooling effect
Note S7. Sorption isotherm
Note S8. True density measurement
Note S9. Water quality test
Note S10. Estimation of 24-hour water production by lab-test experiments
Note S11. Global water production estimation

Part II: Supplementary Figures

Fig. S1. Synthesis of LiCl-cellulose composite.

Fig. S2. Macroscopic and microscopic morphology of LiCl-cellulose composite and cellulose scaffold. (a) Photo images of LiCl-cellulose composite and (b) SEM images of sample fiber. Optical microscopic images of dry (c) and wet at 167% water content (d) cellulose scaffold. The microscale fibers appear more transparent when water occupies the porous space.

Fig. S3. SEM and EDS mapping of 30 wt% LiCl-cellulose composite sample. The corresponding elements are chloride, carbon and oxygen.

Fig. S4. SEM and EDS mapping of 50 wt% LiCl-cellulose composite. (a) SEM show some leakage of LiCl. Zoom-in SEM (b) and corresponding EDS mapping (c) of the leaked LiCl region.

Fig. S5. Tensile test of cellulose scaffold and LiCl-cellulose composite. (a) Tensile strength of cellulose scaffold and LiCl-cellulose composite samples in the dry and wet states. The uncertainty of each sample is the standard deviation with a sample size of three, which is due to the sample variances of dimensions and random errors during the mechanical tests. (b) Strain-stress curves of LiCl-cellulose composite and cellulose scaffold in both wet and dry conditions.

Fig. S6. Optical transmittance. Transmittance in the UV-VIS-NIR range and IR range for the cellulose scaffold and LiCl-cellulose composites.

Fig. S7. Optical reflectance. Reflectance in the UV-VIS-NIR range and IR range for the cellulose scaffold and LiCl-cellulose composites.

Fig. S8. Field test setup. Photo and schematic of temperature measurement setup (a) and cooling power measurement setup (b) in daytime for demonstration.

Fig. S9. Theoretical radiative cooling power. The LiCl-cellulose composite is in wet and dry conditions and cellulose is in dry conditions.

Fig. S10. Lab test schematic. Water uptake characterization in an environmental chamber.
Fig. S11. Water uptake of LiCl-cellulose composite. The sample is at 10, 20, and 30 wt% under different RH with a 9 °C surface temperature drop. The error bars represent the propagated errors from mass measurement.
Fig. S12. Experimental and theoretical sorption isotherm. Theoretical sorption isotherm of cellulose scaffold (a) and LiCl solution (b) is at 290 K and 300 K. (c) Experimental and theoretical sorption isotherm of LiCl-cellulose composite sample at T_a=T_s=298 K.
Fig. S13. Field test demonstration of enhancement by radiative cooling effect. Experimental setup photo (a) and water uptake measured in a field test (b) with and without radiative cooling effects.
Fig. S14. Sorption test under constant conditions for demonstration of synergistic effect. Water uptake of cellulose scaffold, LiCl powder, and LiCl-cellulose composite in the DVS at 0%, 30%, and 70% RH conditions at 25 °C.
Fig. S15. Water harvesting prototype. Solar-driven desorption and recondensation after 1 min (a) and (b) after 10 min (b).
Fig. S16. Water quality test results. Ion concentration in the collected water sample from field test by ICP-MS. The WHO standard of corresponding ions is also plotted.
Fig. S17. Desorption by solar heating or hot plate heating. Photos (a) and water uptake (b) of desorption process by solar heating under 1 sun. (c) Comparison of water production by a solar lamp at 1 sun and hot plate.
Fig. S18. Accelerated cyclic water harvesting tests. Schematic (a) and 100 capture-release cycles (b) of 30 wt% LiCl-cellulose composite at room temperature and accelerated by a nebulizer and a hot plate.
Fig. S19. Sorption isotherm by DDI. Water uptake vs. RH at 25 °C for 10, 20, 30 wt% LiCl-cellulose composites in a DDI method.
Fig. S20. True density of LiCl-cellulose composite as a function of LiCl mass fraction. The uncertainty of each data point is the standard deviation with a sample size of five. The dashed line is a reference obtained by the rule of mixture.

Part III: Supplementary Tables

Table S1. GAB model parameters for cellulose scaffold samples.
Table S2. Parameters for solubility boundaries of LiCl.
Table S3. Empirical parameters for relative vapor pressure π as a function of mass fraction of the salt in solution ξ.
Table S4. Mass weight ratio of water harvesting mechanisms without radiative cooling.
Table S5. Mass weight ratio of water harvesting mechanisms with radiative cooling.

Supplementary Discussions

Note S1. Morphological analysis of LiCl-cellulose composite

Scanning Electron Microscopy (SEM, Quanta) and optical microscopy (OMAX, M837ZL-C180U3) were used to reveal the hierarchical fiber structures of the LiCl-cellulose composite (Fig. S2). Since non-woven cloth has strong mechanical strength and high-water adsorption capability, it is chosen to exemplify the cellulose-based textile and used as the scaffold.

In Fig. S3, carbon and oxygen can be identified in the EDS mapping of the LiCl-cellulose composite since cellulose fibers are biopolymers. The mapped chlorine is mainly from LiCl coating on the fiber surface. Fig. S4 shows the SEM and EDS images of 50 wt% LiCl-cellulose composite. The LiCl leakage was found in the coating film. Higher LiCl content is desirable for the composite sorbent, while the risk of solution leakage may occur during the water sorption process. Thus, one of the challenges in making the stable composite is to choose the optimal LiCl content.

Note S2. Cost analysis of the sample preparation

The utility cost during processes is composed of the water in solution preparation and the electricity of oven drying. We take the 30 wt% LiCl-cellulose composite as an example. On the laboratory scale, the amount of water consumption can be estimated as the amount of water mixing with the amount of LiCl to reach 0.5 mol L^{-1} concentration, which is 0.84 Liter of water. Therefore, the cost of the water consumption is 3.53e-4 – 7.13e-4 USD m^{-2}, given the local water rate as $1.59-3.21 per 1000 gallons of water. The electricity consumption is 1.98 kWh m^{-2} assuming the forced convection oven (220V, 6A) can dry 1 m^2 of composite all at once. The local electricity rate of 0.15 USD kWh^{-1} leads to an electricity cost of 0.297 USD m^{-2}. The total process cost on the lab scale can be estimated as ~0.297 USD m^{-2} since the electricity cost is dominant.

Note S3. Energy consumption estimation for measured water uptake

An estimation can be done by assuming a 9-hour absorption period with an average cooling power of 110 W m^{-2} from Fig. 3d, a water uptake of 4.66 kg kg^{-1} where 40% of the total water uptake is ascribed to sorption. The heat release from sorption is ΔH_{LiCl} = 3536 kJ L^{-1} for LiCl [1] and $\Delta H_{cellulose}$ = 3250 kJ L^{-1} for cellulose [2], respectively. The latent heat for water vapor condensation (ΔH_{vap}) is assumed 2440 kJ L^{-1} at 25 °C indeed counteracts the radiative cooling power. The heat release from sorption and condensation for a 30 wt% LiCl-cellulose composite can be estimated as

$$\begin{aligned}&[(\mathrm{wt\%}_{\mathrm{LiCl}} \cdot \Delta H_{\mathrm{LiCl}} + \mathrm{wt\%}_{\mathrm{cellulose}} \cdot \Delta H_{\mathrm{cellulose}}) \cdot \text{sorption water uptake} \\ &+\Delta H_{\mathrm{vap}} \cdot \text{dew water uptake}] \cdot \rho_{\mathrm{A}} = 775\ \mathrm{kJ\ m^{-2}}\end{aligned} \tag{S1}$$

where ρ_A is the area density. The total cooling energy generated over the 9 hours can be estimated by multiplying the duration and average radiative cooling power as 3564 kJ m^{-2}, which makes the heat of release occupy only 21.7% of the total cooling energy.

Note S4. Theoretical cooling power estimation

The net cooling power (P_{net}) of the sample can be estimated from

$$P_{net} = P_{rad} - P_{conv} - P_{atm} \quad \text{(S2)}$$

where,

P_{rad}: the power density of thermal radiation emitted by the sample,

P_{atm}: the power density of downward thermal radiation from the atmosphere,

P_{conv}: the effective power density loss including convection and conduction from the sample,

There is no power density of solar irradiation term as the experiments were done during the night. P_{rad} is the maximal cooling power available due to radiative cooling and can be derived from the emissivity spectrum of the sample by

$$P_{rad}(T) = \int_0^\infty \varepsilon(\lambda) I_{BB}(T, \lambda) d\lambda \quad \text{(S3)}$$

where I_{BB} is blackbody radiation intensity as a function of emitter temperature according to Planck's law. P_{atm} can be calculated by the amount of power emitted from the atmosphere and absorbed by the sample where the atmospheric emissivity spectrum and the absorptivity spectrum of the cellulose sample are used (Eq. S4). The absorptivity spectrum of the sample equals the emissivity spectrum according to thermal equilibrium and Kirchhoff's radiation law:

$$P_{atm}(T_{atm}) = \int \int_0^\infty \varepsilon(\lambda) \varepsilon_{atm}(\lambda, \theta) I_{BB}(T_{atm}, \lambda) d\lambda d\Omega \quad \text{(S4)}$$

where the angular part of the atmospheric emissivity can be obtained by $\varepsilon_{atm}(\lambda, \theta) = 1 - \tau(\theta)^{1/\cos\theta}$ [3] where $\tau(\theta)$ is the angular atmospheric transmissivity.

The P_{conv} term of the top surface can be evaluated via the sample temperature, ambient temperature, and the effective heat transfer coefficient (Eq. S5):

$$P_{conv} = h(T - T_{amb}) \quad \text{(S5)}$$

where h is the effective heat transfer coefficient and a common value of 15 W m^{-2} K^{-1} is used [4]. The bottom surface of the sample was in contact with an insulated tabletop where its thickness is 35 mm.

Note S5. Composite-water-energy interaction mechanism

The enhanced water capturing performance stems from four mechanisms: 1. Physisorption of cellulose; 2. Chemisorption of salt, forming salt hydrates; 3. Sorption of water vapor into salt solution (here called 1st condensation) if RH reaches the deliquescence point; 4. Condensation of water vapor due to vapor saturation (here called 2nd condensation).

The total water uptake m_{tot} is the sum of all four mechanisms:

$$m_{\mathrm{tot}} = \sum\nolimits_{i=1}^{3} \omega_i m_i (a_{\mathrm{w}}, T) + m_4 \tag{S6}$$

where ω_i, $m_i(a_{\mathrm{w}},T)$ are the mass ratio and water uptake of each mechanism, respectively. The parameter a_w denotes the water activity or relative humidity. It is noted that the cellulose mass ratio is $\omega_1 = 1 - (\omega_2 + \omega_3 + \omega_{\mathrm{intact\_salt}})$. The value of m_4 can be very high because the saturation-induced condensation is only kinetically limited by the cooling power of the cellulose and the heat and mass transfer rate among the sample, the air next to the sample, and the ambient environment.

The multiscale porous structure exposes a large surface area facilitating water harvesting kinetics. Given the thin thickness of the sample and fast sorption kinetics, the material system is assumed to be in equilibrium with the environment. It is also assumed that the air next to the sample has the same temperature as the sample, T_{s}.

Due to radiative cooling, the sample temperature is lower than the ambient temperature ($T_{\mathrm{s}} < T_{\mathrm{a}}$). Consequently, the saturation water vapor pressure near the sample is smaller than that of the ambient conditions, $p_{\mathrm{s}}*(T_{\mathrm{s}}) < p_{\mathrm{a}}*(T_{\mathrm{a}})$.

The water vapor pressure p_{wv} equals the product of RH and saturation vapor pressure p*:

$$p_{\mathrm{wv}} = a_{\mathrm{w}} \times p^*(T) \tag{S7}$$

Therefore, the RH next to the sample is higher than that of the ambient RH, i.e. $a_{\mathrm{w,s}} > a_{\mathrm{w,a}}$.

For physisorption of cellulose (mechanism 1), we employ the Guggenheim-Anderson-de Boer (GAB) sorption model. The GAB model outperforms the BET model as it covers a wider water activity range [5]. The GAB model successfully describes the sorption isotherm of cellulosic materials:

$$m = \frac{W_1 \cdot C \cdot K \cdot a_{\mathrm{w}}}{(1 - K \cdot a_{\mathrm{w}})(1 - K \cdot a_{\mathrm{w}} + CK \cdot a_{\mathrm{w}})} \tag{S8}$$

where m is the moisture content (ratio of the mass of water to mass of dry material). W_1 the moisture content corresponding to the monolayer on the whole free surface. C and K are related to temperature and the heat of adsorption (H) of the monolayer and intermediate layers, respectively. In general C and K should fulfill: $0 < K \leq 1$ and $C > 0$. For $C \geq 2$, the sorption is of type II, i.e. with points of inflection [6].

$$C = C_0 \exp\left(\frac{H_1 - H_n}{T_{\mathrm{amb}}} \right) \tag{S9}$$

$$K = K_0 \exp\left(\frac{H_\mathrm{L} - H_n}{T_\mathrm{amb}}\right) \tag{S10}$$

Where H_1, H_n, and H_L are the heat of sorption of the monolayer, multilayer, and latent heat, respectively. The H_L does not change much within the relevant temperature range. Therefore, a value of 2437 kJ mol^{-1} is used here. Previous reports showed that $H_1 - H_n \approx 4.87$ kJ mol^{-1} [7]. As multilayer adsorbed water resembles bulk liquid water, here we assume $H_n \approx H_\mathrm{L}$ and therefore $K \approx K_0$. The sorption isotherms of cellulose scaffolds are measured using a vapor sorption analyzer.

Table S1. GAB model parameters for cellulose scaffold samples.

Sample	W_1	C	K
Cellulose scaffold 1	0.0666	9.8584	0.0080
Cellulose scaffold 2	0.0655	10.4217	0.0081
Cellulose scaffold 3	0.0692	9.5750	0.0076

It is noted that these values are very close to previous experimental reports of regenerated cellulose films, which had 0.0593, 11.00 and 0.88 for W_1, C and K, respectively [8]. The temperature effect can be manifested by Fig. S12a, where lower temperature results in higher sorption amount m.

The LiCl salt adsorbs a large amount of moisture forming either hydrate or salt solution, as documented by the phase diagram of the LiCl-H_2O system [9]. The solubility boundaries define the conditions at which the crystals and solution co-exist. Above and below the solubility boundaries, the salt exists in solution and hydrate state, correspondingly. The salt hydrate under normal working conditions will be a mixture of LiCl, LiCl·H_2O and LiCl·$2H_2O$. Therefore, the moisture stored in the form of hydrate (mechanism 2) corresponds to a water uptake of 0.42 to 0.85. The water uptake of salt solution (mechanism 3) requires a rather complicated conversion from the phase diagram, as shown below.

The empirical formula of the solubility boundaries is:

$$\varphi \equiv \frac{T}{T_\mathrm{c}} = \sum_{i=0}^{2} A_i \xi^i \tag{S11}$$

where φ and ξ are the reduced temperatures with respect to the critical temperature of water and the mass fraction of the salt in solution, respectively. T_c is the critical temperature of water, i.e. 647.096 K.

Table S2. Parameters for solubility boundaries of LiCl.

	ξ	A_0	A_1	A_2
Ice*	0.000 ~ 0.253	0.422088	-0.09041	-2.93635
LiCl-5H_2O	0.253 ~ 0.287	-0.00534	2.01589	-3.11459
LiCl-3H_2O	0.287 ~ 0.369	-0.56036	4.72308	-5.81105
LiCl-2H_2O	0.369 ~ 0.452	-0.31522	2.88248	-2.62433
LiCl-H_2O	0.452 ~ 0.558	-1.31231	6.17767	-5.03479
LiCl	0.558 ~ 1	-1.3568	3.44854	

*The ice line is $\theta = A_0 + A_1\xi + A_2\xi^{2.5}$.

The relative vapor pressure π can be related to the mass fraction of the salt in solution ξ using the following empirical formula:

$$\pi \equiv \frac{P_{\text{solution}}(\xi, T)}{P_{\text{H}_2\text{O}}(T)} = \pi_{25} f(\xi, \theta) \tag{S12}$$

Where $P_{\text{solution}}(\xi,T)$ and $P_{\text{H}_2\text{O}}(T)$ are the saturation pressure of salt solution and pure water. The empirical variables on the right-hand side of the equation are

$$f(\xi, \theta) = A + B\theta \tag{S13}$$

where

$$A = 2 - \left[1 + \left(\frac{\xi}{\pi_0}\right)^{\pi_1}\right]^{\pi_2}$$

$$B = \left[1 + \left(\frac{\xi}{\pi_3}\right)^{\pi_4}\right]^{\pi_5} - 1$$

$$\pi_{25} = 1 - \left[1 + \left(\frac{\xi}{\pi_6}\right)^{\pi_7}\right]^{\pi_8} - \pi_9 e^{-\frac{(\xi-0.1)^2}{0.005}} \tag{S14}$$

The corresponding parameters are given in Table S3.

Table S3. Empirical parameters for relative vapor pressure π as a function of mass fraction of the salt in solution ξ.

π_0	π_1	π_2	π_3	π_4	π_5	π_6	π_7	π_8	π_9
**0.28**	4.30	0.60	0.21	5.10	0.49	0.362	-4.75	-0.40	0.03

Note that moisture content m and salt mass fraction ξ relate to each other by $m = \frac{1-\xi}{\xi}$.

Using the equations above, the sorption isotherm of salt solution at different temperatures can be obtained, as shown in Fig. S12b using 290 K and 300 K as examples. It is evident that lower temperature facilitates water sorption. The theoretical limit of LiCl sorption can be more than ten times of its dry mass.

To validate the modeling, the sorption isotherms of the composites are measured (sample and ambient temperature both at 298 K, i.e. without radiative cooling). The model agrees with the DVS measurement (Fig. S12c). The deliquescence RH is set to be 11 %. The mass weight ratios of the mechanisms are summarized in Table S4.

Table S4. Mass weight ratio of water harvesting mechanisms without radiative cooling.

**LiCl-cellulose composite**	ω_1	ω_2	ω_3	ω_{intact_salt}
30 wt%	0.7	0.2 to 0.02	0.01 to 0.24	0.09 to 0.04
20 wt%	0.8	0.1 to 0.02	0.01 to 0.17	0.09 to 0.01
10 wt%	0.9	0.08 to 0.001	0.01 to 0.099	0.01 to 0

For the radiative cooling case, sample and ambient temperature are 292 K and 298 K, respectively. For ambient RH_a < 65% ($a_{w,a}$ < 0.65), i.e. RH_s < 100% ($a_{w,s}$ < 1), the water harvest is controlled by the first three mechanisms. The mass weight ratios of the three mechanisms are summarized in Table S5. For RH_a > 65% ($a_{w,a}$ > 0.65), the 2nd water condensation starts which is controlled by the cooling power. The m_4 is estimated to be a linear function of time because the cooling power is constant. It is also noted that, with higher salt content, the chemical potential gradient between the system and air is larger, and consequently the composite harvests water faster.

Table S5. Mass weight ratio of water harvesting mechanisms with radiative cooling.

LiCl-cellulose composite	ω_1	ω_2	ω_3	ω_{intact_salt}	dm_4/dRH
35 wt%	0.65	0	0.25 to 0.35	0.1 to 0	42
25 wt%	0.75	0	0.1 to 0.25	0.25 to 0	40
10 wt%	0.9	0	0.09 to 0.1	0.01 to 0	35

Note S6. Field test of radiative cooling effect

For the comparison of the significance of radiative cooling, the field test started at 12:31 AM and ended at 9:10 AM. A comparative field test was conducted to justify the radiative cooling effect on the AWH performance. A 30 wt% LiCl-cellulose composite sample was divided into two pieces. One piece is exposed to the sky (with radiative cooling) and the other is shielded by suspended cardboard that cut off the pathway of radiative cooling (Fig. S13a). The 47.5% improvement of the water uptake profile by the radiative cooling further verifies the significance of removing heat for AWH (Fig. S13b).

Note S7. Sorption isotherm

DDI measurement

The sorption isotherms of LiCl-cellulose composite are characterized by the Vapor Sorption Analyzer (VSA). The equipment utilizes the dynamic dewpoint isotherm (DDI) method for fast and robust data collection. The traditional dynamic vapor sorption (DVS) method is dependent on the moisture-sample equilibrium that requires the sample to stay at a preset RH for a preset time or until the sample weight change is within tolerance, which may take an infinitely long time. In practice, days and weeks are spent to ensure the equilibrium at each step of RH increment while reducing the time and speeding up may sacrifice measurement accuracy [10]. In contrast, DDI measures the RH (with chilled mirror dewpoint sensor) and sample weight simultaneously. The RH in the sample chamber is always changing, thus the process is dynamic and has no dependence on the moisture-sample equilibrium [11]. The DDI isotherm better resembles reality as the samples will be exposed to constantly changing ambient conditions. The results obtained by DDI will be similar to results from the DVS for the samples tested in this work as both cellulose and LiCl have fast vapor diffusion that allows moisture to penetrate the samples.

SPS measurement

The DVS method is useful to provide information on water absorption as a function of time under any specified RH and temperature. To analyze the material synergistic effect without radiative cooling, the cellulose, LiCl, and LiCl-cellulose composite are tested simultaneously by the Projekt Messtechnik SPS moisture sorption analyzer. The derivative of the curves in Fig. S14 gives the rate of the change of the water uptake in Fig. 4f.

Note S8. True density measurement

The dashed line in Fig. S20 is calculated based on the rule of mixture, i.e. $\rho_{\text{compsoite}} = \text{wt\%}_{\text{cellulose}}\ \rho_{\text{cellulose}} + \text{wt\%}_{\text{LiCl}}\ \rho_{\text{LiCl}}$, as a reference. The true density increases proportionally with the increase of LiCl mass fraction, and the trend well matches the rule of mixture. The measured true densities are used in specific surface characterization by BET.

Note S9. Water quality test

Water quality is crucial for the development of atmospheric water harvesting. The water release process by solar heating is similar to evaporation and distillation. Therefore, the recondensed water is separated from the LiCl-cellulose composite, and LiCl is not expected in harvested water. An element analysis is conducted on the water harvested from the developed prototype by inductively coupled plasma mass spectrometry (ICP-MS). Fig. S16 shows the concentration of Lithium, Magnesium, potassium, and Arsenic ions. Compared to the World Health Organization (WHO) standard, the concentrations of different elements are within the safety limit. The existence of Li^+ ions indicates inevitable contamination when manually opening and collecting water from the prototype, which can be improved when the composite material is applied to a more comprehensive and automated system. The health guideline for Li^+ concentration in drinking water has not been advised by WHO, but the magnitude of the Li^+ concentration of our water sample is one order smaller than the other works [12,13]. The results indicate the cellulose material's capability of generating safe and clean water.

Note S10. Estimation of 24-hour water production by lab-test experiments

Desorption kinetics and energy are also key factors that affect the daily water production of the AWH material.

First, the LiCl-cellulose composite with 30 wt% LiCl loading underwent water adsorption at 30-90% RH for 2 hours in the controlled chamber (dT = 9 °C). After the adsorption process in the environmental chamber, the composite was transferred to a petri dish with the bottom covered by a dark substrate. The desorption process in the lab was simulated by a solar simulator with Xenon Arc Lamp (UXL-150, Newport 67005). The solar simulator emitted 1 sun (1 kW m^{-2}) of irradiation. The dark substrate absorbed the sunlight and generated heat to facilitate the removal of the moisture from the saturated composite. The mass of the composite was measured at a time interval between 5-15 minutes until 99% of captured water was released to determine the desorption time (Fig. S17a). Fig. S17b shows the water production and required desorption time for 30-90% RH.

Alternatively, desorption can also be achieved by an active heat source. Here, we used a hotplate at 80 °C to release the absorbed water and compared the desorption kinetics between the LiCl-cellulose composite and LiCl powders. The two samples started with the same amount of absorbed water. The mass of each sample was recorded every ~10 minutes. The 24-hour water production curves were also estimated for comparison (Fig. S17c). The desorption rate by a solar lamp at 1 sun is faster compared to the continuous water production by the hotplate, which is due to the direct heating by solar heating versus large heat loss through conduction by hotplate heating.

Note S11. Global water production estimation

The hourly data for the year 2021 was collected from the database of the National Centers for Environmental Prediction (NCEP) including RH, ambient temperature, and total cloud coverage. Each day has four data points for each variable. The water production rate was expressed as a piecewise function of relative humidity based on the 24 hours multicycle water collection rate at an ambient temperature of 25 °C and dT=9 °C between RH of 30-90%. At above 60% RH range, the water production rate was assumed to linearly vary with RH; while between 30-60% RH, the water production rate was fitted with an exponential function with an R^2 value of 0.997 and was extrapolated down to 8% RH. The ambient temperature and RH from the database were standardized to equivalent RH at 25 °C by their equal absolute humidity and ideal gas equation.

$$AH = \frac{p_{\mathrm{wv}}}{R_{\mathrm{w}}T}, \quad \varphi_{25} = \frac{AH \cdot R_{\mathrm{w}} \cdot T_{25}}{p^{*}(T_{25})} \tag{S15}$$

where AH is the absolute humidity, φ_{25} is the standardized relative humidity at 25 °C, and R_{w} is the specific gas constant of water vapor. T_{25} is input as the temperature in Kelvin at 25 °C. The vapor pressure at given conditions can be calculated based on Eq. S16 with saturated vapor pressure and RH. p^* is determined from

$$\ln\left(\frac{p^{*}}{p_c}\right) = \left(\frac{T_c}{T}\right)(b_1\vartheta + b_2\vartheta^{1.5} + b_3\vartheta^{3} + b_4\vartheta^{3.5} + b_5\vartheta^{4} + b_6\vartheta^{7.5}) \tag{S16}$$

Where the p_{c}, T_{c}, and ϑ are critical pressure, temperature of water, and transformed temperature (1-T/T_{c}). The coefficients b_1 to b_6 can be found in Ref. [14].

The cloud coverage was assumed to linearly impact the water production rate, i.e. with 100% cloud coverage, the water production rate reduced to 50% of the original value, and with 0% cloud coverage, it remained original value.

$$M = \begin{cases} 6.5712 + 2.7857e^{\frac{\varphi_{25}-30}{9.3748}} & 8\% \le \varphi_{25} < 60\% \\ 2.926(\varphi_{25} - 60) + 74.916 & \varphi_{25} \ge 60\% \\ 0 & \text{Otherwise} \end{cases} \tag{S17}$$

where φ_{25} is plugged in as percentage and M means the water production rate in the unit of L kg^{-1} day^{-1}.

Supplementary Figures

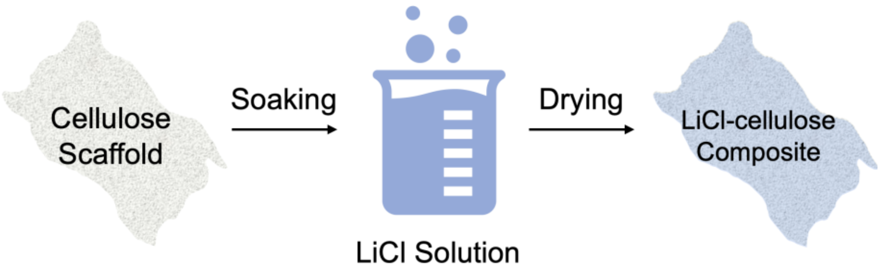

Fig. S1. Synthesis of LiCl-cellulose composite.

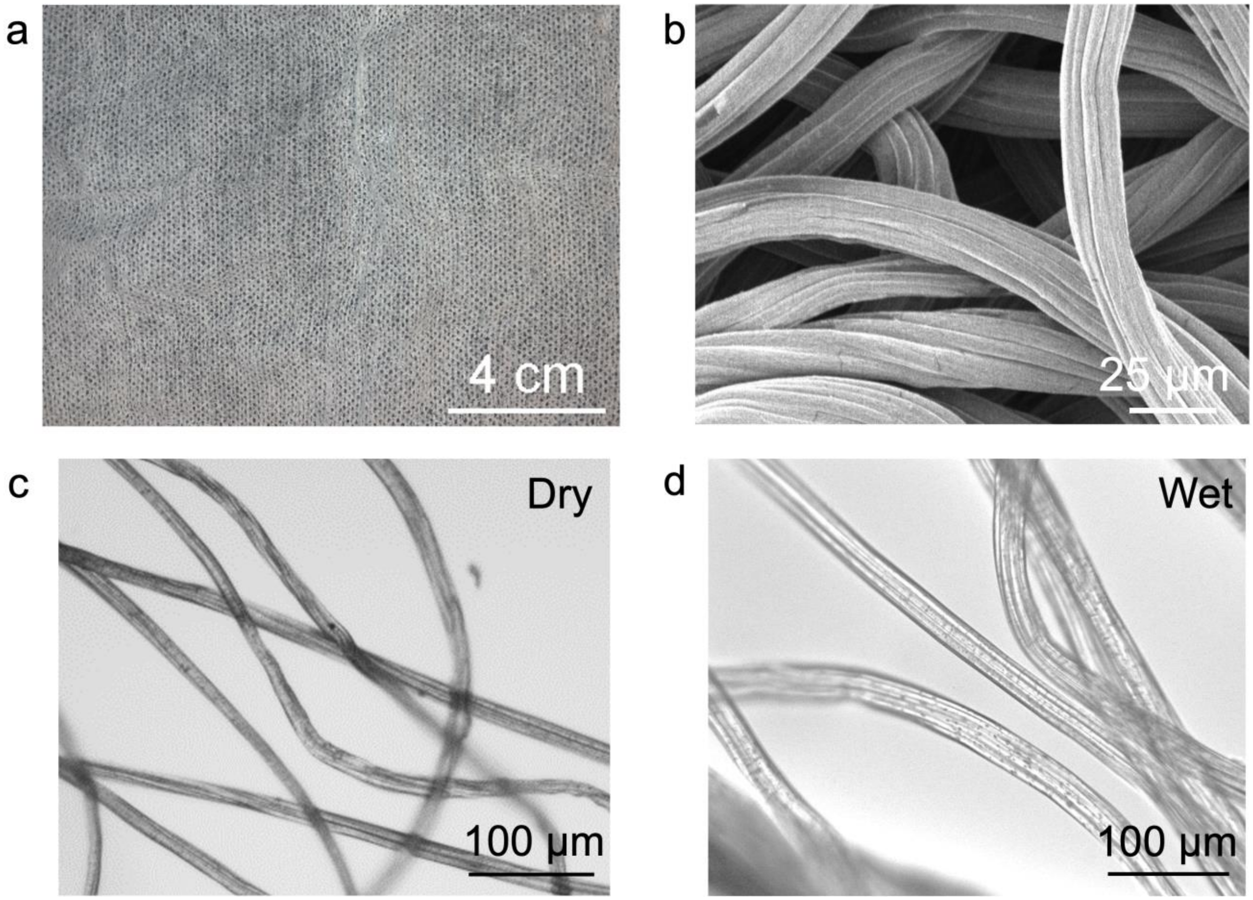

Fig. S2. Macroscopic and microscopic morphology of LiCl-cellulose composite and cellulose scaffold. (a) Photo images of LiCl-cellulose composite and (b) SEM images of sample fiber. Optical microscopic images of dry (c) and wet at 167% water content (d) cellulose scaffold. The microscale fibers appear more transparent when water occupies the porous space.

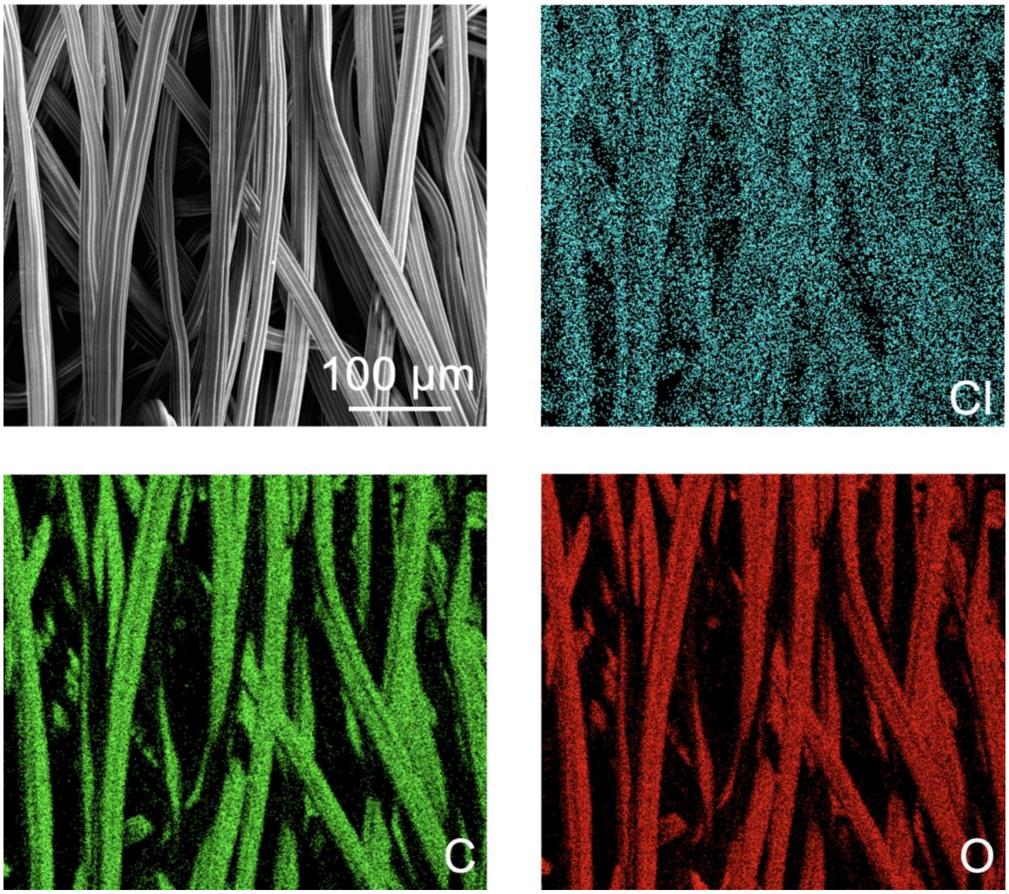

Fig. S3. SEM and EDS mapping of 30 wt% LiCl-cellulose composite sample. The corresponding elements are chloride, carbon and oxygen.

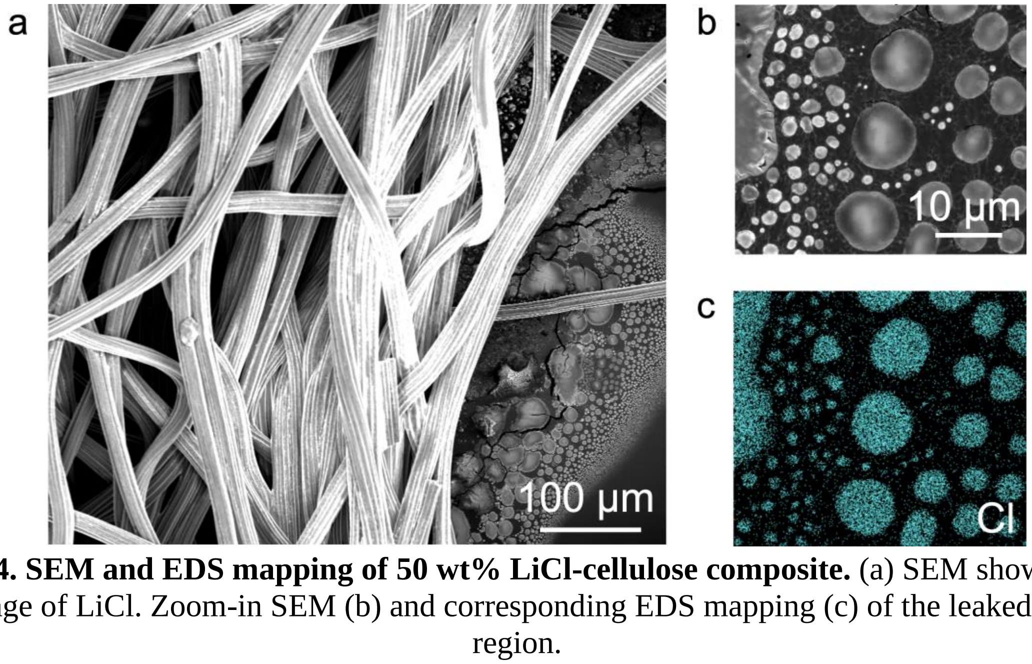

Fig. S4. SEM and EDS mapping of 50 wt% LiCl-cellulose composite. (a) SEM show some leakage of LiCl. Zoom-in SEM (b) and corresponding EDS mapping (c) of the leaked LiCl region.

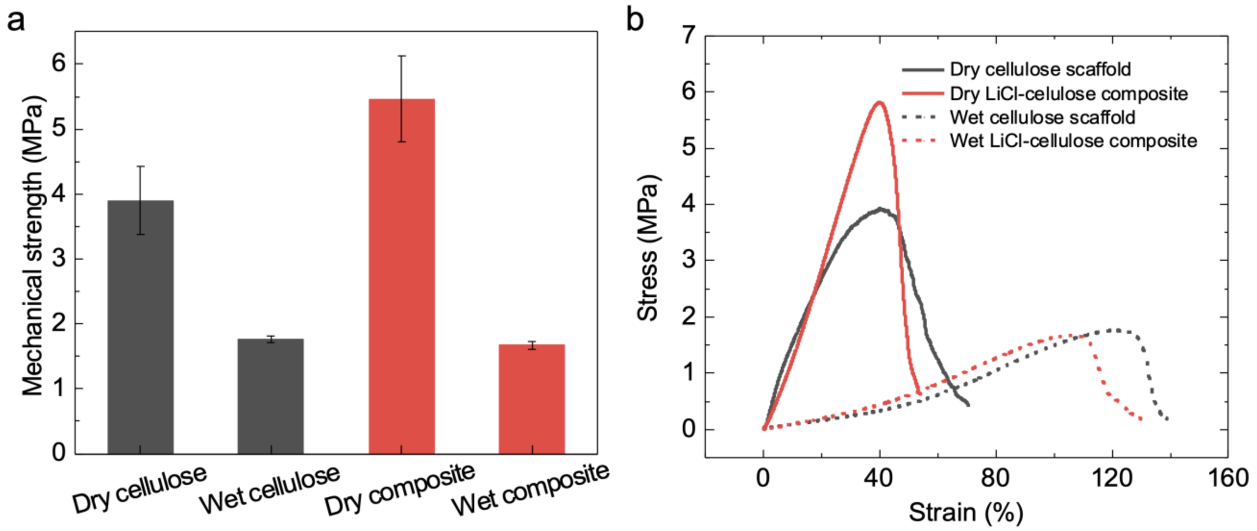

Fig. S5. Tensile test of cellulose scaffold and LiCl-cellulose composite. (a) Tensile strength of cellulose scaffold and LiCl-cellulose composite samples in the dry and wet states. The uncertainty of each sample is the standard deviation with a sample size of three, which is due to the sample variances of dimensions and random errors during the mechanical tests. (b) Strain-stress curves of LiCl-cellulose composite and cellulose scaffold in both wet and dry conditions.

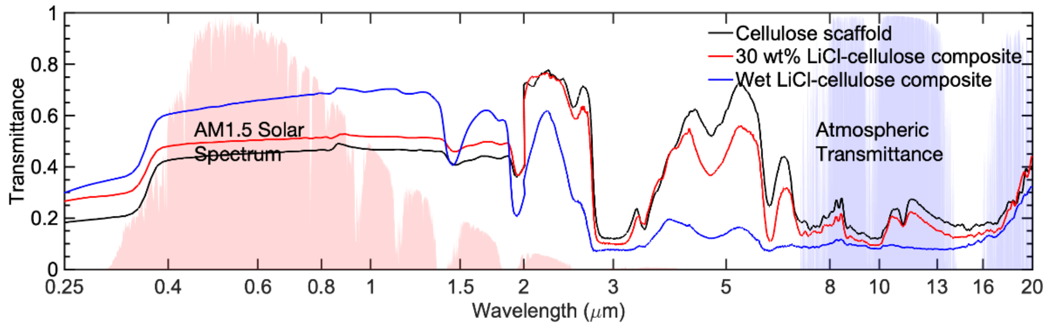

Fig. S6. Optical transmittance. Transmittance in the UV-VIS-NIR range and IR range for the cellulose scaffold and LiCl-cellulose composites.

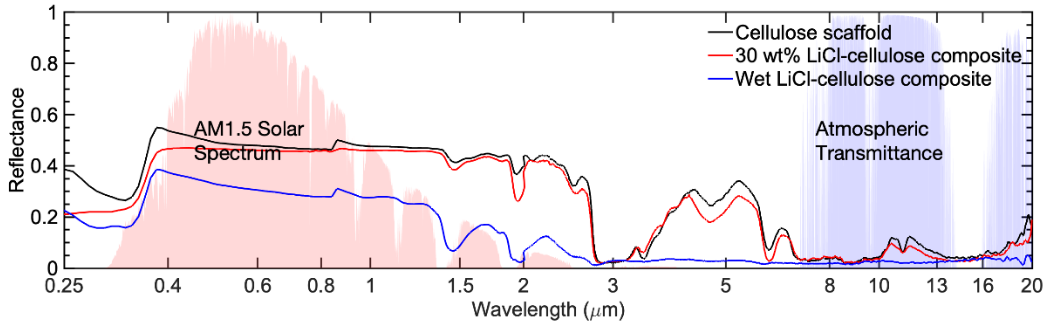

Fig. S7. Optical reflectance. Reflectance in the UV-VIS-NIR range and IR range for the cellulose scaffold and LiCl-cellulose composites.

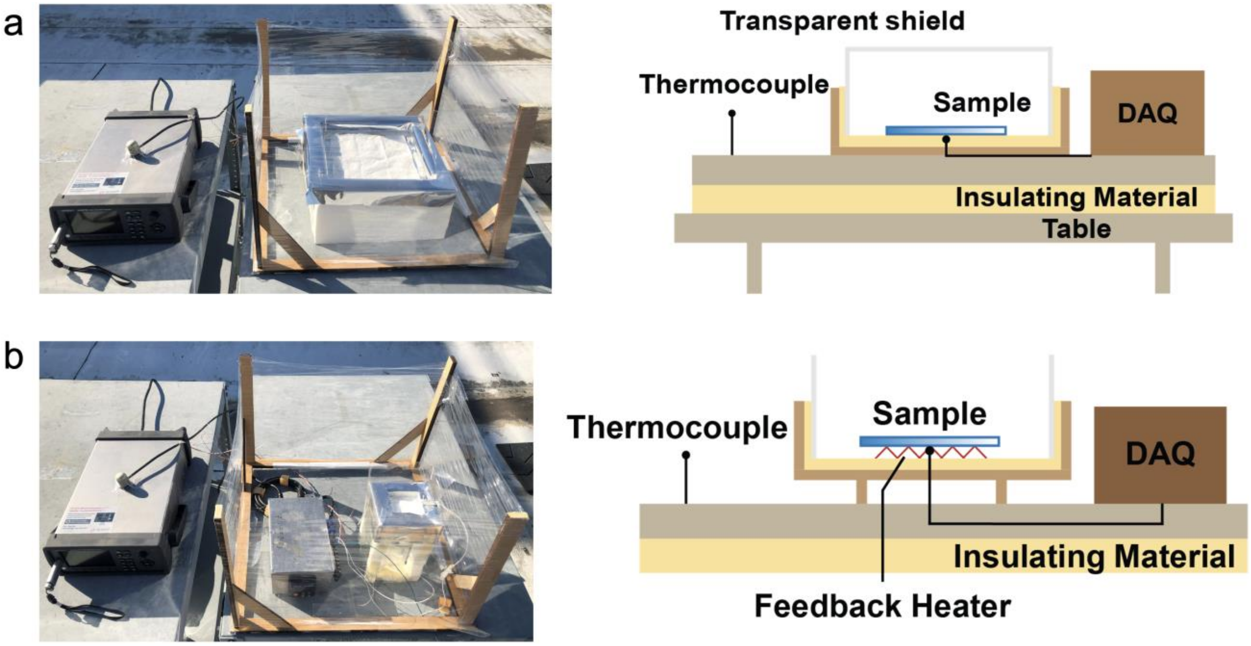

Fig. S8. Field test setup. Photo and schematic of temperature measurement setup (a) and cooling power measurement setup (b) in daytime for demonstration.

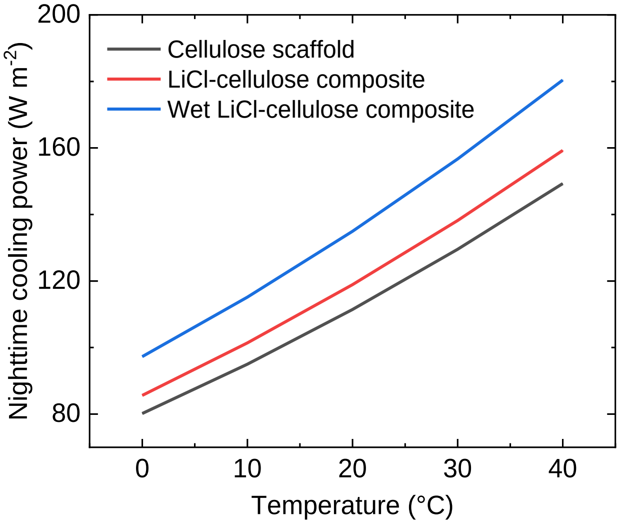

Fig. S9. Theoretical radiative cooling power. The LiCl-cellulose composite is in wet and dry conditions and cellulose is in dry conditions.

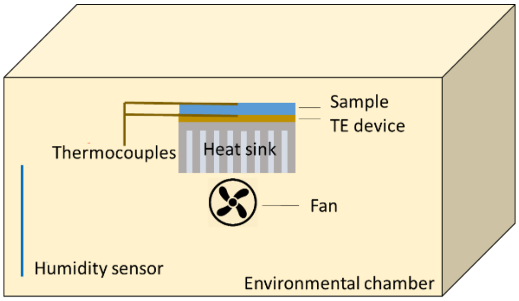

Fig. S10. Lab test schematic. Water uptake characterization in an environmental chamber.

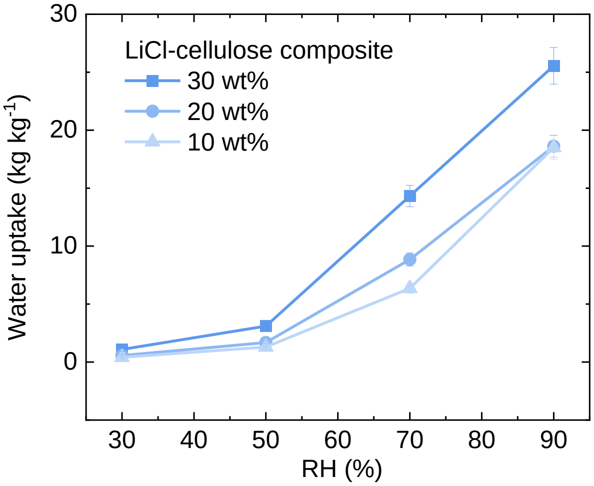

Fig. S11. Water uptake of LiCl-cellulose composite. The sample is at 10, 20, and 30 wt% under different RH with a 9 °C surface temperature drop. The error bars represent the propagated errors from mass measurement.

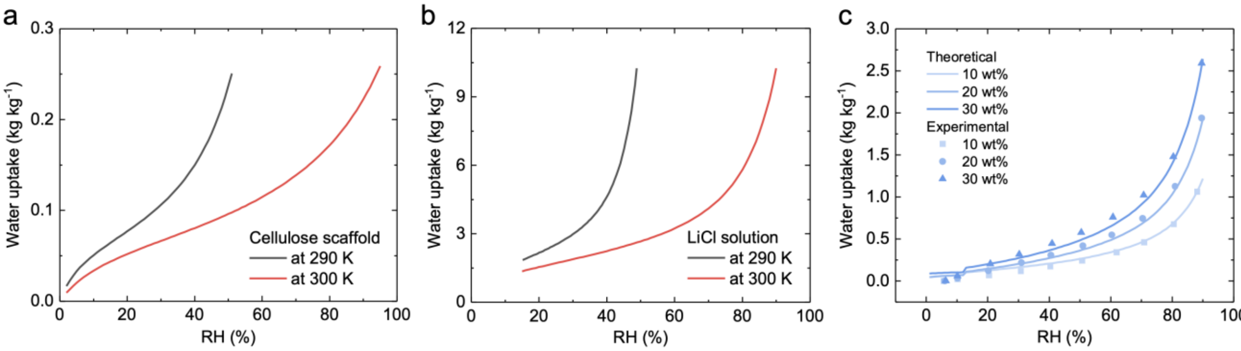

Fig. S12. Experimental and theoretical sorption isotherm. Theoretical sorption isotherm of cellulose scaffold (a) and LiCl solution (b) is at 290 K and 300 K. (c) Experimental and theoretical sorption isotherm of LiCl-cellulose composite sample at T_a=T_s=298 K.

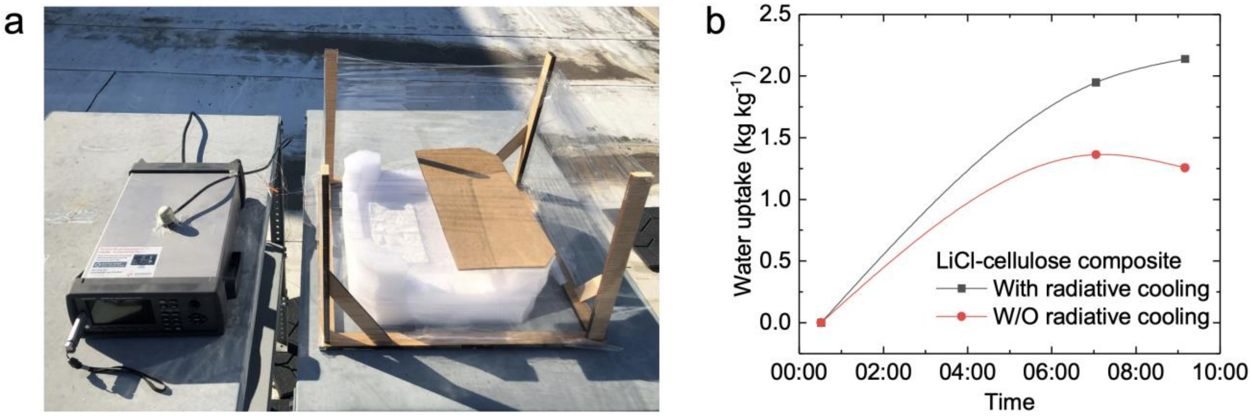

Fig. S13. Field test demonstration of enhancement by radiative cooling effect. Experimental setup photo (a) and water uptake measured in a field test (b) with and without radiative cooling effects.

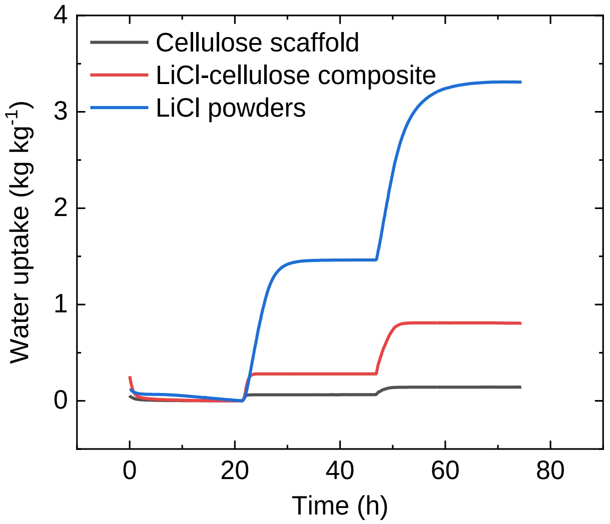

Fig. S14. Sorption test under constant conditions for demonstration of synergistic effect. Water uptake of cellulose scaffold, LiCl powder, and LiCl-cellulose composite in the DVS at 0%, 30%, and 70% RH conditions at 25 °C.

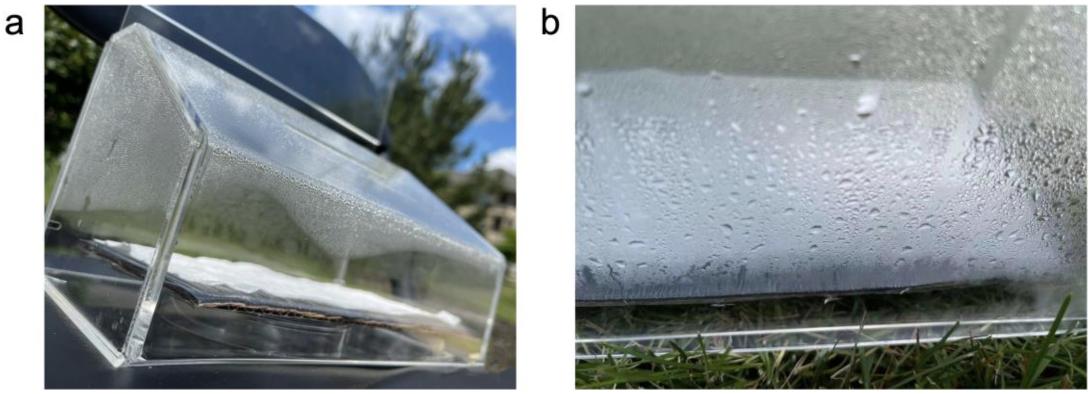

Fig. S15. Water harvesting prototype. Solar-driven desorption and recondensation after 1 min (a) and (b) after 10 min (b).

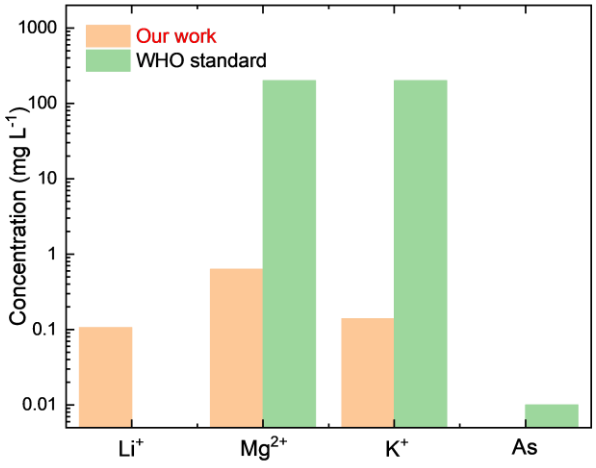

Fig. S16. Water quality test results. Ion concentration in the collected water sample from field test by ICP-MS. The WHO standard of corresponding ions is also plotted.

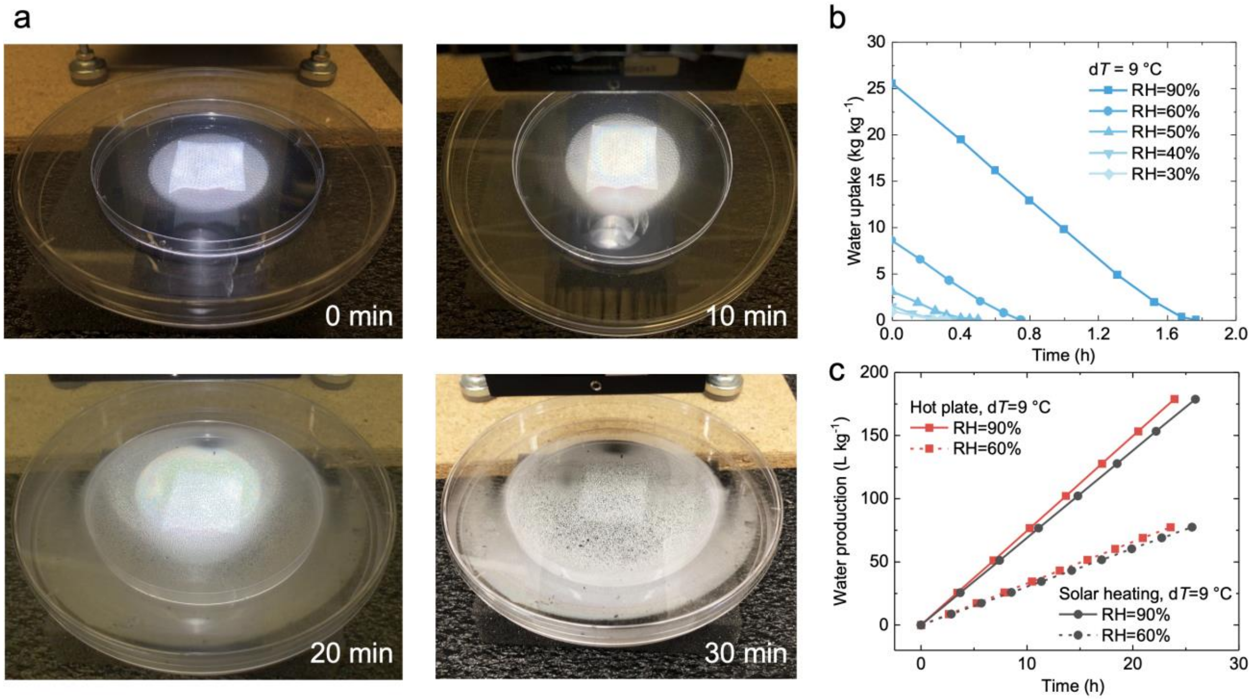

Fig. S17. Desorption by solar heating or hot plate heating. Photos (a) and water uptake (b) of desorption process by solar heating under 1 sun. (c) Comparison of water production by a solar lamp at 1 sun and hot plate.

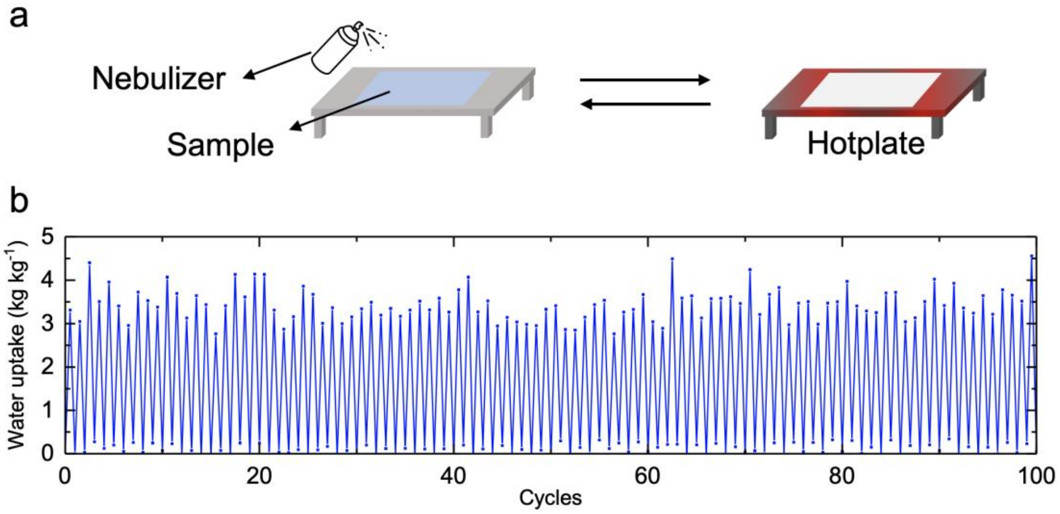

Fig. S18. Accelerated cyclic water harvesting tests. Schematic (a) and 100 capture-release cycles (b) of 30 wt% LiCl-cellulose composite at room temperature and accelerated by a nebulizer and a hot plate.

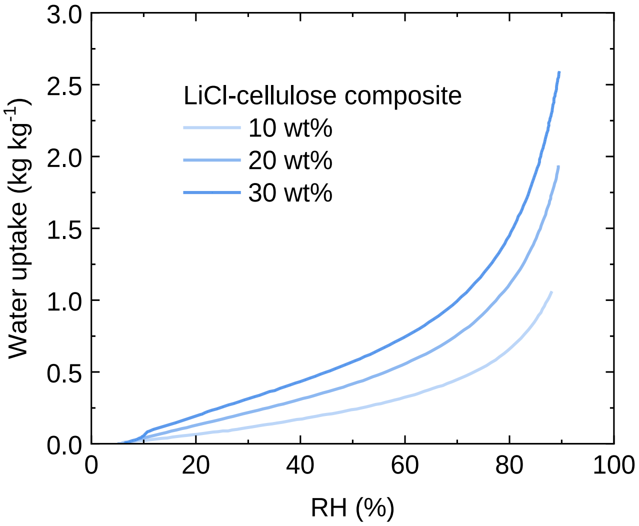

Fig. S19. Sorption isotherm by DDI. Water uptake vs. RH at 25 °C for 10, 20, 30 wt% LiCl-cellulose composites in a DDI method.

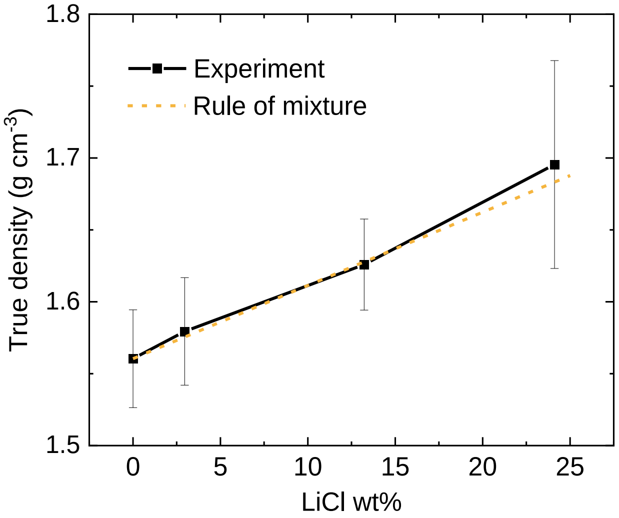

Fig. S20. True density of LiCl-cellulose composite as a function of LiCl mass fraction. The uncertainty of each data point is the standard deviation with a sample size of five. The dashed line is a reference obtained by the rule of mixture.

Supplementary References

1 Rau, J. J. *Thermodynamic characteristics of lithium chloride in rotary heat and mass exchangers*, (1989).

2 NES, B., HAL, K. & AR, N. H. Heat–moisture interactions and phase change in fibrous material. *Thermal and Moisture Transport in Fibrous Materials*, 424 (2006).

3 Tikhonravov, A. V., Trubetskov, M. K. & DeBell, G. W. Application of the needle optimization technique to the design of optical coatings. *Applied optics* **35**, 5493-5508 (1996).

4 Liu, C., Fan, J. & Bao, H. Hydrophilic radiative cooler for direct water condensation in humid weather. *Solar Energy Materials and Solar Cells* **216**, 110700 (2020). https://doi.org:https://doi.org/10.1016/j.solmat.2020.110700

5 Timmermann, E. O. Multilayer sorption parameters: BET or GAB values? *Colloids and Surfaces A: Physicochemical and Engineering Aspects* **220**, 235-260 (2003).

6 Blahovec, J. Sorption isotherms in materials of biological origin mathematical and physical approach. *Journal of Food Engineering* **65**, 489-495 (2004).

7 Grunin, Y. B., Grunin, L. Y., Schiraya, V. Y., Ivanova, M. S. & Masas, D. S. Cellulose–water system's state analysis by proton nuclear magnetic resonance and sorption measurements. *Bioresources and Bioprocessing* **7**, 1-11 (2020).

8 Cazón, P., Vázquez, M. & Velázquez, G. Regenerated cellulose films with chitosan and polyvinyl alcohol: Effect of the moisture content on the barrier, mechanical and optical properties. *Carbohydrate polymers* **236**, 116031 (2020).

9 Conde, M. R. Properties of aqueous solutions of lithium and calcium chlorides: formulations for use in air conditioning equipment design. *International Journal of Thermal Sciences* **43**, 367-382 (2004).

10 Carter, B. & Fontana, A. Dynamic dewpoint isotherm verses other moisture sorption isotherm methods. *Application note. Decagon Devices, Pullman, WA* **210** (2008).

11 Schmidt, S. J. & Lee, J. W. Comparison between water vapor sorption isotherms obtained using the new dynamic dewpoint isotherm method and those obtained using the standard saturated salt slurry method. *International Journal of Food Properties* **15**, 236-248 (2012).

12 Shan, H. *et al.* High-yield solar-driven atmospheric water harvesting with ultra-high salt content composites encapsulated in porous membrane. *Cell Reports Physical Science* **2**, 100664 (2021).

13 Wang, J. *et al.* High-yield and scalable water harvesting of honeycomb hygroscopic polymer driven by natural sunlight. *Cell Reports Physical Science* **3**, 100954 (2022).

14 Wagner, W. & Pruß, A. The IAPWS formulation 1995 for the thermodynamic properties of ordinary water substance for general and scientific use. *Journal of physical and chemical reference data* **31**, 387-535 (2002).
